# Computational modeling and thermal analysis of magnetized nanofluid flow with physio-chemical interaction and chemical reaction between two non-parallel walls

**DOI:** 10.3389/fchem.2025.1466356

**Published:** 2025-05-15

**Authors:** Shahid Sohail, Zahir Shah, Muhammad Rooman, Waris Khan, Mansoor H. Alshehri, Narcisa Vrinceanu, Elisabeta Antonescu

**Affiliations:** ^1^ Department of Mathematical Sciences, University of Lakki Marwat, Lakki Marwat, Khyber Pakhtunkhwa, Pakistan; ^2^ Department of Mathematics, Hazara University, Mansehra, Khyber Pakhtunkhwa, Pakistan; ^3^ Department of Mathematics, College of Science, King Saud University, Riyadh, Saudi Arabia; ^4^ Department of Industrial Machines and Equipments, Faculty of Engineering, “Lucian Blaga” University of Sibiu, Sibiu, Romania; ^5^ Preclinical Department Faculty of Medicine, Lucian Blaga University of Sibiu, Sibiu, Romania

**Keywords:** nanomaterials, magnetohydrodynamic, non-parallel walls, nanofluid, heat transfer efficiency, chemical component

## Abstract

The study of how energy undergoes changes in physio-chemical interactions involving Al_2_O_3_ and γ-Al_2_O_3_ with water and C_2_H_6_O_2_ within converging and diverging channels is of great significance, given its potential applications in today’s advanced technology. We have used two types of oxide nanoparticles, namely, Al_2_O_3_ and γ- Al_2_O_3_, with water and C_2_H_6_O_2_. The purpose of this study is to investigate an innovative comparative magnetohydrodynamic (MHD) nanofluid flow and heat transport with the impact of thermal radiation on water and ethylene glycol (EG) suspended with Al_2_O_3_ and γ-Al_2_O_3_ nanoparticles. A novel comparison of concentration of Al_2_O_3_–H2O, γ-Al_2_O_3_–H_2_O, and γ Al_2_O_3_–C_2_H_6_O_2_ nanofluids is investigated under the influence of chemical reactions. The system of nonlinear ordinary differential equations was obtained via a similarity transformation and then solved using the homotopy analysis method (HAM) in Mathematica. The temperature and velocity profiles are obtained numerically for a range of controlling parameter values, including the volume percentage φ of nanomaterials, the magnetic effect parameter M, the radiation parameter Rd, and Eckert number Ec in convergent/divergent channels. The concentration profiles of Al_2_O_3_–H_2_O, γ-Al_2_O_3_–H_2_O, and γ-Al_2_O_3_–C_2_H_6_O_2_ tri-nanofluids are calculated numerically for governing parameter values, including those accounting for chemical reactions. The investigation’s findings indicate that there is greater heat transport in γ-Al_2_O_3_–C_2_H_6_O_2_ and γ-Al_2_O_3_–H_2_O than in Al_2_O_3_–H_2_O. We have demonstrated that there is good agreement between the current results and those found in the literature for various values of the magnetic field parameter, thermal radiation parameter, and nanoparticle volume fraction.

## 1 Introduction

In the realm of thermo science and thermal engineering, substantial advancements have been made in the pursuit of enhancing heat transfer. One notable approach involves the incorporation of additives into liquids. This strategy becomes particularly relevant when the inherent characteristics of the flowing fluids themselves pose constraints on the efficiency of heat transfer. To address this, solid additives are introduced into the base liquids, effectively modifying the properties of transport, dynamics of flow, and thermal transmission characteristics of these fluids (Zimparov, 2002). In addition to detailing the historical progression within this particular field, Hetsroni and Rozenblit (1994) conducted a study focusing on the thermal interplay between a turbulent flow containing particles and a heated plate. Their research centered on a liquid–solid blend comprising water and polystyrene particles.

Nanofluids, which consist of colloidal suspensions of nanoscale particles distributed steadily and intermittently into traditional fluids, were developed as a solution to the primary issue of heat transfer augmentation. This newly created category is known as nanofluids. The efficiency of energy transport in nanofluids is influenced by both the characteristics and size of the nanoscale particles, along with the volume percentage of solids. Several experimental studies have highlighted that nanofluids exhibit significantly higher thermal conductivity than conventional pure fluids (Wang et al., 1999; Lee et al., 1999; Xuan and Li, 2003). These findings suggest that nanofluids hold substantial potential for enhancing heat transfer processes. In contrast to traditional methods of improving heat transfer, which involve adding millimeter- or micrometer-sized particles to fluids, nanofluids are expected to be an optimal choice for practical applications. This is due to the minuscule size of the nanoparticles, which allows nanofluids to behave much like pure fluids, resulting in minimal or negligible increases in pressure drop. Choi and Eastman (1995) made an earlier contribution in this direction. Later, Eastman at el. (2001) showed that ethanol glycol-based copper nanofluid has higher thermal conductivity than pure ethanol glycol. Xuan and Li (2003) experimentally showed that Cu–water nanofluid can enhance the heat transfer process compared to pure-based liquid. The magnetohydrodynamic (MHD) fluid flow and heat transfer of hybrid nanofluid under thermal radiation were investigated by Chamkha et al. (2019), who developed a model for nanofluids that incorporates the Roseland distribution estimate, Brownian motion, and thermophoresis, expanding the understanding of these complex phenomena. Mohamed and Wahid (Abdel-wahed, 2017) investigated the effects of nonlinear thermal radiation and magnetic fields on nanofluid flow and temperature over a moving surface.

Hybrid nanofluids represent a specialized category within the broader domain of nanofluids, characterized by the deliberate blending of two or more distinct nanoparticle types into a base fluid. Nanofluids, in their general definition, encompass finely dispersed nanoparticles at the nanometer scale, entrained within a base fluid, which can be either a liquid or a gas. Notably, hybrid nanofluids derive their nomenclature from their unique composition, whereas multiple nanoparticle variants coexist within the same base fluid. The engineering of hybrid nanofluids is oriented toward imbuing them with distinctive and tailored properties, rendering them particularly suitable for a diverse array of applications. Of paramount significance among these applications are domains such as heat transfer, cooling systems, and energy generation, where the exigencies of thermal management and energy efficiency are critical. Numerous scholarly investigations have delved into the realm of hybrid nanofluids, each offering unique perspectives and findings. Notably, Hanif et al. (2020a) provided a complete entropy analysis, focusing on unsteady mixed convection in a magneto-hybrid nanofluid flowing over an inverted cone enclosed by a porous media. Khan and Rasheed (2021) investigated thermal properties in detail using silicon dioxide and molybdenum disulfide in a three-dimensional heated surface framework. Furthermore, Hanif et al. (2021) studied the effectiveness of hybrid nanofluids in the presence of heat radiation—specifically, in a heated cone under the influence of a magnetic field. The numerical analysis was performed using the Crank–Nicolson method. Hanif et al. (2020b) used numerical analysis to account for varying viscosities in water-based hybrid nanofluid flows around an inverted permeable cone by incorporating the effects of a magnetic field and radiative heat flux. Saqib et al. (2019) investigated the velocity and temperature of a hybrid nanofluid with nanoparticles of alumina and copper. They used Laplace transformation and fractional Caputo–Fabrizio derivative to calculate their outcomes.

The MHD, often called hydromagnetics or magnetofluid dynamics, studies fluid dynamics in electrically conducting mediums. The MHD field can be traced back to the pioneering work of Hannes Alfvén, a luminary in plasma physics who was awarded the Nobel Prize in Physics in 1970 for his efforts. The core assumption of magnetohydrodynamics is that magnetic fields can create electric currents within a flowing, conductive fluid. Consequently, these induced currents engender forces within the fluid, which reciprocally lead to alterations in the magnetic field itself, shaping the fundamental dynamics of MHD. The application of MHD on the nanofluid flow can alter its behavior. Keeping in view the significance of the MHD application, it has attracted the interest of many researchers. In several boundary value issues, researchers have employed the MHD flow with stretched surfaces to enhance thermal performance. Nourazar et al. (2017) investigated one such case. When different nanoparticles were suspended in a base fluid, they studied the flow of a nanofluid. When the magnetic parameter increases, they discovered an increase in the temperature profile. Babu et al. (2018) explained the nanofluid flow issue under the influence of heat radiation, where the MHD flow was induced by stretching a sheet. Venkateswarlu and Narayana (2019) presented the MHD flow across a porous flat plate and used slip effects to analyze the resulting model’s thermo-physical properties. Dehghani et al. (2019) investigated water–Al_2_O_3_ nanofluid mixed convection in a grooved channel with internal heat production in solid cylinders using numerical modeling of magneto hydrodynamics (MHD. The influence of a non-uniform magnetic field on the ferrofluid flow and heat transfer in plain and wavy channels was investigated by Mousavi et al. (2020). They discovered that the channel with wavy walls had a stronger magnetic field impact on the flow field and heat transfer than the channel with plain walls.

Thermal radiation, often referred to as heat radiation, entails the emission of electromagnetic waves in the form of heat by an object owing to its temperature. This phenomenon holds a fundamental status within the realms of physics and engineering, assuming a pivotal function across diverse domains, encompassing heat transfer, energy systems, and materials science. The extensive use of thermal radiation in the domains of physics and engineering, particularly concerning the design of mechanical components, spacecraft technology, and gas turbine systems, has bestowed paramount significance upon it. England and Emery (1969) investigated the impact of thermal radiation on a vertical flat plate with the laminar-free convection boundary layer flow for both non-absorbing and absorbing gases. Furthermore, Reddy (2019) investigated unsteady MHD-free convection flow through an isothermal porous vertical plate. They used the numerical technique Ritz finite element method to solve their problem. Their finding showed that velocity and temperature reduced for higher radiation parameters. Samad and Mansur-Rahman (1970) investigated unsteady MHD free convection flow with the influence of thermal radiation and chemical reactions through an infinite isothermal porous vertical plate. They also used the numerical technique known as the Ritz finite element method. They found that the velocity profile was enhanced by large thermal and mass Grashof numbers, while the velocity profile decreased for large magnetic parameters. Roja (2022) studied magnetohydrodynamic Powell–Eyring hybrid nanofluid flow in a vertical porous channel, accounting for porous media effects, magnetism, convective circumstances, dissipation energy, heat generation, and radiation. Their findings show that entropy generation increases with the Grashof number, Biot number, and radiation parameter but decreases with magnetism, Darcy number, and Eyring–Powell factors. The data revealed that increased radiation was associated with a decrease in liquid temperature and an increase in entropy. Rashad et al. (2023) studied MHD Cu–Fe_3_O_4_/ethylene glycol (EG) nanofluids in a permeable media using the Powell–Eyring fluid model. Their findings indicated that increasing both the radiation and magnetic field increased the fluid’s velocity. On the other hand, it was discovered that the radiation effect alone increased the temperature of the boundary layer. Furthermore, many investigators have studied how radiation affects the transfer of heat. These include analyses of Cu/H_2_O laminar flows in vertical channels (Mostafazadeh et al., 2019), MHD flows of Cu/H_2_O amongst parallel plates (Dogonchi et al., 2016), and MHD flows of GO/H_2_O in permeable channels for nanofluids (Dogonchi et al., 2017). Additionally, investigations encompassed MHD flows of CuO–Al_2_O_3_/H_2_O across two different geometries (Ashwinkumar et al., 2021), MHD flows of Al_2_O_3_–Ag/H_2_O over a stretching sheet (Shoaib et al., 2020), and MHD flows of Williamson MoS_2_–ZnO/EG over a permeable stretching sheet for hybrid nanofluids (Yahya et al., 2021).

The interaction of heat and mass transfer in conjunction with chemical reactions is a highly significant area of study, garnering substantial interest in recent times. This multidisciplinary field finds applications across various industries, including the design of chemical processing equipment, safeguarding crops from freezing damage, food processing, and the operation of cooling towers. The mechanism of an unsteady flow across an infinite vertical plate with a constant heat and mass transfer was studied by Das et al. (1994) in relation to the impact of the first-order homogeneous chemical reaction. The impact of the chemical reaction and injection on the flow characteristics in an unstable upward motion of an isothermal plate was explored by Muthucumaraswamy and Ganesan (2001). Chamkha (2003) investigated MHD flow over a uniformly stretched vertical permeable surface in the presence of heat generation/absorption and a chemical reaction. Rahman and Sattar (2005) investigated the MHD convective flow of micropolar fluid via a rotating vertical porous plate when heat production and/or absorption were present. The analytical solution for an unstable MHD-free convection flow via a semi-infinite vertical permeable moving plate with a heat source and chemical reaction was discovered by Ibrahim et al. (2008). Rahman et al. (2009a) investigated heat transport over a non-stretching sheet in a micropolar fluid with temperature-dependent fluid characteristics. Rahman et al. (2010) and Rahman et al. (2009b) studied heat transmission in micropolar fluid down an inclined plate with varying fluid characteristics under various boundary conditions. Recently, Akbarzadeh (2018) and Yadav (2019) used linear stability analysis to investigate the impact of chemical reactions on convective instability in a horizontal layer of nanofluids. They noticed that increasing the chemical reaction parameter (K_1_) increases the system’s stability.

Radiative nanofluid flow between two non-parallel walls is one of the significant research motivations. This flow is also called the Jeffery–Hamel flow or flow amongst converging/diverging channels. The Jeffrey–Hamel flow is a specific type of non-parallel flow that occurs between two walls, with a gap that changes steadily along the direction of the fluid flow. This flow has significantly enhanced our understanding of non-parallel flows, boundary layer behavior, and turbulence phenomena. Its significance spans multiple fields, from applied research in microfluidics and numerical simulations to basic research in fluid dynamics. It is relevant to many biological, environmental, and engineering applications. It facilitates improvements in heat transfer, drag reduction, mixing, turbulence modeling, and the comprehension of natural processes, which eventually result in increased effectiveness, performance, and sustainability across a range of industries. Alharbi and Adnan (2022) presented a unique heat transfer model for two types of nanofluids and two host solvents. They found that the nanofluids based on Al_2_O_3_ and C_2_H_6_O_2_ have significantly higher conductivity than other nanofluids. Adnan et al. (2022) investigated the energy storage efficiency of tri-hybrid nanofluids (Al_2_O_3_–CuO–Cu/H_2_O) and hybrid nanofluids (Al_2_O_3_–CuO–H_2_O), taking into account the effects of unique viscous dissipation mechanisms. In another study, Adnan et al. (2023) investigated the ternary hybrid nanofluid (Al_2_O_3_–CuO–Fe_3_O_4_)/C_2_H_6_O_2_ flow, with a viscous dissipation effect in slippery converging/diverging channels. They found that ternary hybrid nanofluid has superior heat transfer characteristics compared to simple hybrid nanofluid. Boudjemline et al. (2023) considered the pressure-driven flow of an expanding tube containing a non-Newtonian Oldroyd-B nanofluid. Rawat et al. (2023) investigated a hybrid nanofluid flow in a Darcy–Forchheimer porous medium between two rotating discs. They emphasized the importance of non-uniform heat source/sink and the Cattaneo–Christov model in comprehending the complex flow behavior. Most relevant research studies are as follows: Adnan (2022), Gaffar et al. (2018), Grigore et al. (2017), Pirvut et al. (2018), Sudhagar et al. (2017), Sumithra et al. (2023), Aich et al. (2023), Rushi Kumar et al. (2023), Ismail et al. (2024), and Khan et al. (2024). Abdelsalam et al. (2023a), Abdelsalam et al. (2023b), Abdelsalam and Zaher (2023), El Koumy et al. (2012), Abdelsalam and Bhatti (2023), and Abdelsalam et al. (2024) investigated nanofluid flow, considering different nanoparticles with rheological properties by applying various mathematical models in different geometries.

In the current work, we consider a novel comparison of heat transfer and fluid velocity in water and EG dispersed by Al_2_O_3_ and γ Al_2_O_3_ nanoparticles between converging and diverging channels under MHD and thermal radiation effects. We also investigate novel comparative concentration profiles for Al_2_O_3_–H_2_O, γ -Al_2_O_3_–H_2_O, and Al_2_O_3_–EG under the impact of chemical reactions and the Schmidt number (Sc).

It is critical to respond to the following questions in this study:

• Heat transfer mechanism comparison in Al_2_O_3_–H_2_O, γ -Al_2_O_3_–H_2_O, and Al_2_O_3_–EG.
• MHD and solid volume fraction effects on fluid velocity between converging and diverging channels.
• Effects of MHD and thermal radiation on fluid temperature between converging and diverging channels.
• Effects of chemical reaction on the concentration of the nanoparticles used.
• The behavior of the Sherwood number in Al_2_O_3_–H_2_O, γ -Al_2_O_3_–H_2_O, and Al_2_O_3_–EG.

## 2 Problem formulations

### 2.1 Physical modeling

The study examines the incompressible flow of various nanofluids (Al_2_O_3_–H_2_O, γ -Al_2_O_3_–H_2_O, and Al_2_O_3_–EG) in polar coordinates between two inclined non-parallel walls. Along the center, the fluid flows at the same speed. Only the u component drives the motion of the nanoliquid since the flow only occurs along the center line. Furthermore, θ represents the polar angle, and r represents the direction in radials. The conduit walls remain motionless and are set at an angle of separation denoted by *α*, which indicates convergence for α<0 and divergence for α>0 . Moreover, we consider that in the z-direction, there is no magnetic field. Further assumptions are detailed as follows:

• Steady, incompressible, 2D but unidirectional flow of various nanofluids (Al_2_O_3_–H_2_O, γ -Al_2_O_3_–H_2_O, and Al_2_O_3_–EG) are considered.
• For the flow analysis through the channel, the cylindrical polar coordinates $\left(r,\theta ,z\right)$ are taken.
• A varying magnetic field is acting normally in the radial direction.
• Thermally conducting nanofluids (Al_2_O_3_–H_2_O, γ -Al_2_O_3_–H_2_O, and Al_2_O_3_–EG) are considered.

**FIGURE 1:**
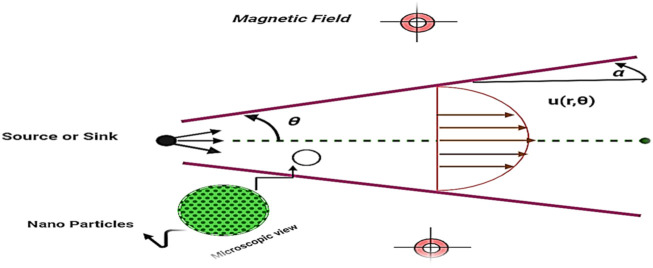
(Geometry of the problem): Flow of nanofluids in the presence of MHD and thermal radiation between two non-parallel walls.

The flow of nanofluid between two non-parallel barriers is shown in Figure 1.

### 2.2 Mathematical modeling

#### 2.2.1 Conservation equations for nanofluids

The continuity equation, the Navier–Stokes equations, and concentration equation in polar coordinates are as follows (Abdelsalam et al., 2023b; Abdelsalam and Zaher, 2023; El Koumy et al., 2012):

$$\frac{1}{r}\frac{\partial \left(ru\right)}{\partial r}=0,$$ (1)

$$\rho_{nf}\left(u\frac{\partial u}{\partial r}\right)=-\frac{\partial p}{\partial r}+\mu_{nf}\left(\frac{\partial^{2}u}{\partial r^{2}}+\frac{1}{r}\frac{\partial u}{\partial r}+\frac{1}{r^{2}}\frac{\partial^{2}u}{\partial \theta^{2}}-\frac{1}{r^{2}}u\right)-\frac{\sigma_{nf}B_{{}^{\circ}}^{2}u}{r^{2}},$$ (2)

$$-\frac{1}{r\rho_{nf}}\frac{\partial p}{\partial \theta }+\frac{2\mu_{nf}}{r^{2}\rho_{nf}}\frac{\partial u}{\partial \theta }=0,$$ (3)

$${\left(\rho C_{p}\right)}_{nf}u\frac{\partial T}{\partial r}=k_{nf}\left(\frac{\partial^{2}T}{\partial r^{2}}+\frac{1}{r}\frac{\partial T}{\partial r}+\frac{1}{r^{2}}\frac{\partial^{2}T}{\partial \theta^{2}}\right)+\sigma_{nf}B_{0}^{2}u^{2}+\frac{16\sigma_{\text{nf}}T_{\infty }^{3}}{3k^{*}}\left(\frac{\partial^{2}T}{\partial r^{2}}+\frac{1}{r}\frac{\partial T}{\partial r}+\frac{1}{r^{2}}\frac{\partial^{2}T}{\partial \theta^{2}}\right),$$ (4)

$$u\frac{\partial C}{\partial r}=D_{B}\left(\frac{\partial^{2}C}{\partial r^{2}}+\frac{1}{r}\frac{\partial C}{\partial r}+\frac{1}{r^{2}}\frac{\partial^{2}C}{\partial \theta^{2}}\right)-K_{r}C.$$ (5)

Here, Equation 1 represents the law of conservation of mass. Equations 2–4 represent the equation of momentum and equation for energy, correspondingly. Equation 5 represents the concentration field.

## 3 Thermo-physical properties

The thermo-physical characteristics of nanofluids are inherently contingent upon the thermo-physical attributes of both nanoparticles and base fluids. In the context of our ongoing nanofluid research, we consider the specific heat capacity, density, thermal conductivity, and electrical conductivity of the base fluids and particles. The pertinent values for these thermo-physical properties are presented in Table 1. Table 2 presents the shape factor, sphericity, and shape of the nanomaterials. The nanofluid models and their empirical correlations are provided in Tables 3, 4.

**TABLE 1:** Thermo-physical properties of base fluids and materials (Abdelsalam and Bhatti, 2023; Abdelsalam et al., 2024; Dogonchi and Ganji, 2016).

Model	$\rho \left(Kg/m^{3}\right)$	$c_{p}\left({kg}^{-1}K^{-1}\right)$	σsm	$k\left({Wm}^{-1}K^{-1}\right)$	Pr	Shape
$H_{2}O$	997.1	4,179	$5.5\times 10^{-6}$	0.613	6.96	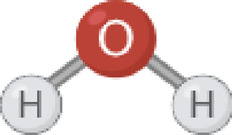
$C_{2}H_{6}O_{2}$	1,116.6	2,382	$4.3\times 10^{-5}$	0.249	204	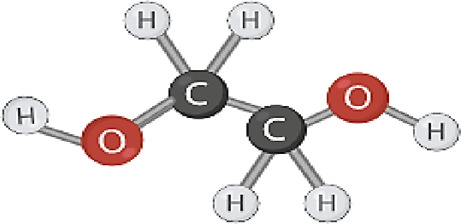
${Al}_{2}O_{3}$	3,970	765	$35\times 10^{6}$	40	…….	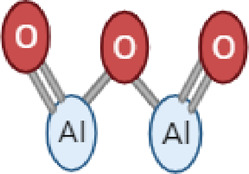

**TABLE 2:** Attributes for different shape factors (Adnan et al., 2023; Boudjemline et al., 2023; Adnan, 2022; Gaffar et al., 2018).

Nanomaterial’s shape	Shape factor m	Sphericity ∈	Shape
Brick	3.7	0.811	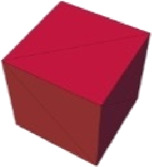
Cylinder	4.9	0.625	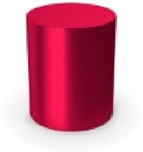
Sphere	3.0	1.000	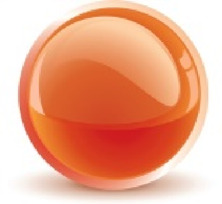
Platelet	5.7	0.526	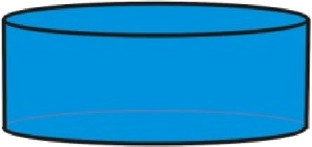
Blade	8.6	---	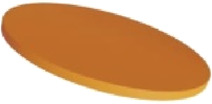
Tetrahedron	4.06	---	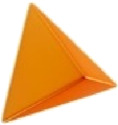
Hexahedron	3.72		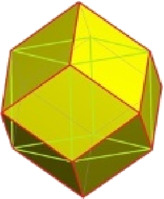

**TABLE 3:** Nanofluid empirical correlation used in the models (Dogonchi and Ganji, 2016; Alharbi and Adnan, 2022; Vishnu Ganesh et al., 2016; Gao et al., 2022).

${Al}_{2}O_{3}-H_{2}O\;and\;{Al}_{2}O_{3}-C_{2}H_{6}O_{2}$	$\gamma {Al}_{2}O_{3}-H_{2}O$		$\gamma {Al}_{2}O_{3}-C_{2}H_{6}O_{2}$
$\mu_{nf=}\mu_{f}{\left(1-\phi \right)}^{-2.5}$	$\mu_{nf=}\mu_{f}\left(123\phi^{2}+7.3\phi +1\right)$		$\mu_{nf=}\mu_{f}\left(306\phi^{2}-0.19\phi +1\right)$
$k_{nf}=k_{f}\left\{\frac{k_{s}+2k_{f}-2\phi \;\left(k_{f}-k_{s}\right)}{k_{s}+2k_{f}+2\phi \;\left(k_{f}-k_{s}\right)}\right\}$	$k_{nf=}k_{f}\left(4.97\phi^{2}+2.72\phi +1\right)$		$k_{nf=}k_{f}\left(28.90\phi^{2}+2.8272\phi +1\right)$
Heat capacity and density			
$\rho_{nf=}\left\{\left(1-\phi \right)+\frac{\phi \rho_{s}}{\rho_{f}}\;\right\}\rho_{f}$		${\left(\rho c_{p}\right)}_{nf}=\left\{\left(1-\phi \right)+\frac{\phi {\left(\rho c_{p}\right)}_{s}}{{\left(\rho c_{p}\right)}_{f}}\;\right\}{\left(\rho c_{p}\right)}_{f}$	

**TABLE 4:** Mathematical expressions for thermo-physical properties for nanofluid (NF) (Dogonchi and Ganji, 2016; Alharbi and Adnan, 2022; Vishnu Ganesh et al., 2016; Gao et al., 2022).

Property	Nanofluid
Density	$\rho_{nf}=\left(1-\phi_{1}\right)\times \rho_{f}+\phi_{1}\rho_{s_{1}}$
Dynamic viscosity	$\mu_{nf}=\frac{\mu_{f}}{{\left[1-\phi_{1}\right]}^{2.5}}$
Heat capacity	${\left(\rho C_{p}\right)}_{nf}=\left[1-\phi_{1}\right]{\left(\rho c_{p}\right)}_{f}+{\phi_{1}\left(\rho c_{p}\right)}_{s_{1}}$
Thermal expansion	${\left(\gamma \right)}_{nf}=\left(1-\phi_{1}\right)\gamma_{f}+\phi_{1}\gamma_{s_{1}}$
Thermal conductivity	$\frac{k_{nf}}{k_{f}}=\frac{k_{s_{1}}+\left(n-1\right)k_{f}-\left(n-1\right)\phi_{1}\left(k_{f}-k_{s_{1}}\right)}{k_{s_{1}}+\left(n-1\right)k_{f}+\phi_{1}\left(k_{f}-k_{s_{1}}\right)}$
Electrical conductivity	$\frac{\sigma_{nf}}{\sigma_{f}}=\frac{\sigma_{s_{1}}+\left(n-1\right)\sigma_{f}-\left(n-1\right)\phi_{1}\left(\sigma_{f}-\sigma_{s_{1}}\right)}{\sigma_{s_{1}}+\left(n-1\right)\sigma_{f}+\phi_{1}\left(\sigma_{f}-\sigma_{s_{1}}\right)}$

### 3.1 Boundary conditions

The feasible boundary conditions for this model are as follows:

$$\left.\begin{array}{l} \frac{\partial u}{\partial \theta }=0\\ \frac{\partial T}{\partial \theta }=0\\ u=U \end{array}\right\},$$ (6)

$$\left.\begin{array}{c} u=0\\ T=T_{W} \end{array}\right\}.$$ (7)

## 4 Non-dimensionalization

We use similarity transformation to reduce the given modeled equation. The appropriate transformations of similarity are specified as (Abdelsalam and Bhatti, 2023; Abdelsalam et al., 2024; Dogonchi and Ganji, 2016)

$$F\left(\eta \right)=\frac{f\left(\theta \right)}{f_{\max}},\theta \left(\eta \right)=\frac{T}{T_{w}},\gamma \left(\eta \right)=\frac{C}{C_{w}},and\;\eta =\frac{\theta }{\alpha }.$$ (8)

Integrating Equation 1, we obtain Equation 9 as follows:

$$f\left(\theta \right)=\text{ru}\left(r,\theta \right).$$ (9)

### 4.1 Final version of the model equations

By using the empirically derived correlations for the nanofluids and incorporating self-similar variables as defined in Equation 8, the flow model becomes nonlinear and coupled.

The initial and boundary conditions in the dimensionless form for the current problem can be articulated as follows:

$$\begin{array}{c} \left.\begin{array}{c} F\left(\eta \right)=1\\ F^{ˊ}\left(\eta \right)=0 \end{array}\right\}\text{ at }\eta =0\\ \begin{array}{c} F\left(\eta \right)=0\text{ at }\eta =1\\ \theta^{ˊ}\left(\eta \right)=0\text{ at }\eta =0\\ \theta \left(\eta \right)=1\text{ at }\eta =1 \end{array} \end{array}.$$ (19)

### 4.2 Physical parameters

The physical parameters used in the modeled equations are explained in the following section, along with their symbols and mathematical forms.

## 5 Quantities of engineering interest

### 5.1 Calculation of the coefficient of skin friction $\left(C_{f}\right)$

Surface drag forces result when fluids are in motion; such forces are characterized by the coefficient factor of friction $\left(Cf_{x}\right)$ . It is defined in Equation 20:

$$Cf_{x}=\frac{\tau_{w}}{\rho_{tnf}U_{w}^{2}}.$$ (20)

The terms $\tau_{w},\rho_{f}$ , and $u_{w}$ represent the wall shear stress, fluid density, and the fluid stretching velocity, respectively. The coefficients of skin friction for the used nanofluids are calculated as given in Equations 21–23:

**Table:** 

${Al}_{2}O_{3}-H_{2}O\;and\;{Al}_{2}O_{3}-C_{2}H_{6}O_{2}$	$$F^{ˊˊˊ}+\left[\left(1-\phi \right)+\frac{\phi \rho_{s}}{\rho_{f}}\right]{\left(1-\phi \right)}^{2.5}2\alpha R_{e}FF^{ˊ}+4\alpha^{2}F^{\prime }-\frac{\sigma_{nf}}{\sigma_{f}}{\left(1-\phi \right)}^{2.5}MF^{ˊ}=$$ (10)
	$${\left(\left\{\frac{k_{s}+2k_{f}-2\phi \;\left(k_{f}-k_{s}\right)}{k_{s}+2k_{f}+2\phi \;\left(k_{f}-k_{s}\right)}\right\}+Rd\right)\theta }^{\prime \prime }+\frac{\sigma_{nf}}{\sigma_{f}}\alpha \left(PrEc\right)MF^{2}=0$$ (11)
	$$\gamma^{\prime \prime }+ScK_{1}\gamma =0$$ (12)
$\gamma {Al}_{2}O_{3}-H_{2}O$	$$F^{ˊˊˊ}+\frac{\left[\left(1-\phi \right)+\frac{\phi \rho_{s}}{\rho_{f}}\right]}{\left(123\phi^{2}+7.3\phi +1\right)\;}2\alpha R_{e}FF^{ˊ}+4\alpha^{2}F^{\prime }-\frac{\sigma_{nf}}{\sigma_{f}}\frac{1}{\left(123\phi^{2}+7.3\phi +1\right)}MF^{ˊ}=0$$ (13)
	$${\left(\left(4.97\phi^{2}+2.72\phi +1\right)+Rd\right)\theta }^{\prime \prime }+\frac{\sigma_{nf}}{\sigma_{f}}\alpha \left(PrEc\right)MF^{2}=0$$ (14)
	$$\gamma^{\prime \prime }+ScK_{1}\gamma =0$$ (15)
$\gamma {Al}_{2}O_{3}-C_{2}H_{6}O_{2}$	$$F^{ˊˊˊ}+\frac{\left[\left(1-\phi \right)+\frac{\phi \rho_{s}}{\rho_{f}}\right]}{\left(306\phi^{2}-0.19\phi +1\right)\;}2\alpha R_{e}FF^{ˊ}+4\alpha^{2}F^{\prime }-\frac{\sigma_{nf}}{\sigma_{f}}\frac{1}{\left(306\phi^{2}-0.19\phi +1\right)\;}MF^{ˊ}=0$$ (16)
	$${\left(\left(28.90\phi^{2}+2.8272\phi +1\right)+Rd\right)\theta }^{\prime \prime }+\frac{\sigma_{nf}}{\sigma_{f}}\alpha \left(PrEc\right)MF^{2}=0$$ (17)
	$$\gamma^{\prime \prime }+ScK_{1}\gamma =0$$ (18)

### 5.2 Nusselt number $\left(Nu\right)$

The Nusselt number $\left(Nu\right)$ characterizes local heat conduction and convective heat transfer at a fixed position, relating heat transfer coefficients to thermal conductivity. The term $\left(N_{ux}\right)$ is formally defined in Equation 24 as follows:

$$Nu=\frac{Q_{w}x}{k_{tnf}\Delta T_{w}}.$$ (24)

The local Nusselt numbers $\left(Nu\right)$ for given nanofluids are calculated as given in Equations 25–27 as follows:

**Table:** 

Parameter	Symbol	Mathematical Expression	Range of the parameters
Reynolds number	Re	$\text{Re}=\frac{\alpha u_{c}}{v_{f}}$	0.1≤Re≤2
Prandtl number	Pr	$\mathrm{Pr}=\frac{\mu_{f}C_{p}}{K_{f}}$	6.9≤Pr≤9
Eckert number	Ec	$Ec=\frac{u_{c}^{2}}{{C_{p}T}_{w}}$	0.1≤Ec≤4
Radiation parameter	Rd	$Rd=\frac{16\sigma_{f\;}T_{\infty }^{3}}{3K^{*}K_{f}}$	0.1≤Rd≤2
Magnetic parameter	M	$M=B_{{}^{\circ}}\sqrt{\frac{\sigma_{f}}{\mu_{f}}}$	0.1≤M≤12.1
Schmidt number	Sc	$Sc=\frac{v_{f}}{D_{B}}$	0.1≤Sc≤2
Chemical reaction parameter	$K_{1}$	$K_{1}=\frac{\alpha^{2}K_{r}}{v_{f}}$	$0.1\leq K_{1}\leq 0.4$

### 5.3 Calculation of the Sherwood number (Sh)

In the case of a mass transfer, the analogous Sherwood number $\left(Sh_{x}\right)$ provides a convective diffusion description similar to that used for heat transfer, involving mass transfer coefficient values and species diffusivity terms. $Sh_{x}$ is defined in Equation 28 as follows:

$$Sh_{x}=\frac{Q_{m}x}{D_{B}\Delta C_{w}}.$$ (28)

The Sherwood numbers for the used nanofluids are calculated as given in Equations 29–31 as follows:

**Table:** 

Nanofluid	Coefficient of skin friction ( $C_{f}$ )
${Al}_{2}O_{3}-H_{2}O\;and\;{Al}_{2}O_{3}-C_{2}H_{6}O_{2}$	$$ReC_{f}=\frac{1}{{\left(1-\phi \right)}^{2.5}\left\{\left(1-\phi \right)+\frac{\phi \rho_{s}}{\rho_{f}}\;\right\}}F^{ˊ}\left(1\right).$$ (21)
$\gamma {Al}_{2}O_{3}-H_{2}O$	$$ReC_{f}=\frac{\left(123\phi^{2}+7.3\phi +1\right)}{\left\{\left(1-\phi \right)+\frac{\phi \rho_{s}}{\rho_{f}}\;\right\}}F^{ˊ}\left(1\right).$$ (22)
$\gamma {Al}_{2}O_{3}-C_{2}H_{6}O_{2}$	$$ReC_{f}=\frac{\left(306\phi^{2}-0.19\phi +1\right)\;}{\left\{\left(1-\phi \right)+\frac{\phi \rho_{s}}{\rho_{f}}\;\right\}}F^{ˊ}\left(1\right).$$ (23)

**Table:** 

Nanofluid	Local Nusselt number
${Al}_{2}O_{3}-H_{2}O\;and\;{Al}_{2}O_{3}-C_{2}H_{6}O_{2}$	$$\alpha Nu=\left\{\frac{k_{s}+2k_{f}-2\phi \;\left(k_{f}-k_{s}\right)}{k_{s}+2k_{f}+2\phi \;\left(k_{f}-k_{s}\right)}\right\}\left({-\theta }^{ˊ}\left(1\right)\right).$$ (25)
$\gamma {Al}_{2}O_{3}-H_{2}O$	$$\alpha Nu=\left(4.97\phi^{2}+2.72\phi +1\right)\left({-\theta }^{ˊ}\left(1\right)\right).$$ (26)
$\gamma {Al}_{2}O_{3}-C_{2}H_{6}O_{2}$	$$\alpha Nu=\left(28.90\phi^{2}+2.8272\phi +1\right)\left({-\theta }^{ˊ}\left(1\right)\right).$$ (27)

**Table:** 

Nanofluid	Sherwood number (Sh)
${Al}_{2}O_{3}-H_{2}O\;and\;{Al}_{2}O_{3}-C_{2}H_{6}O_{2}$	$$Sh=\left\{\frac{k_{s}+2k_{f}-2\phi \;\left(k_{f}-k_{s}\right)}{k_{s}+2k_{f}+2\phi \;\left(k_{f}-k_{s}\right)}\right\}\left({-\gamma }^{ˊ}\left(1\right)\right).$$ (29)
$\gamma {Al}_{2}O_{3}-H_{2}O$	$$Sh=\left(4.97\phi^{2}+2.72\phi +1\right)\left({-\gamma }^{ˊ}\left(1\right)\right)$$ (30)
$\gamma {Al}_{2}O_{3}-C_{2}H_{6}O_{2}$	$$Sh=\left(28.90\phi^{2}+2.8272\phi +1\right)\left({-\gamma }^{ˊ}\left(1\right)\right).$$ (31)

## 6 Solution methodology

**FIGURE 2:**
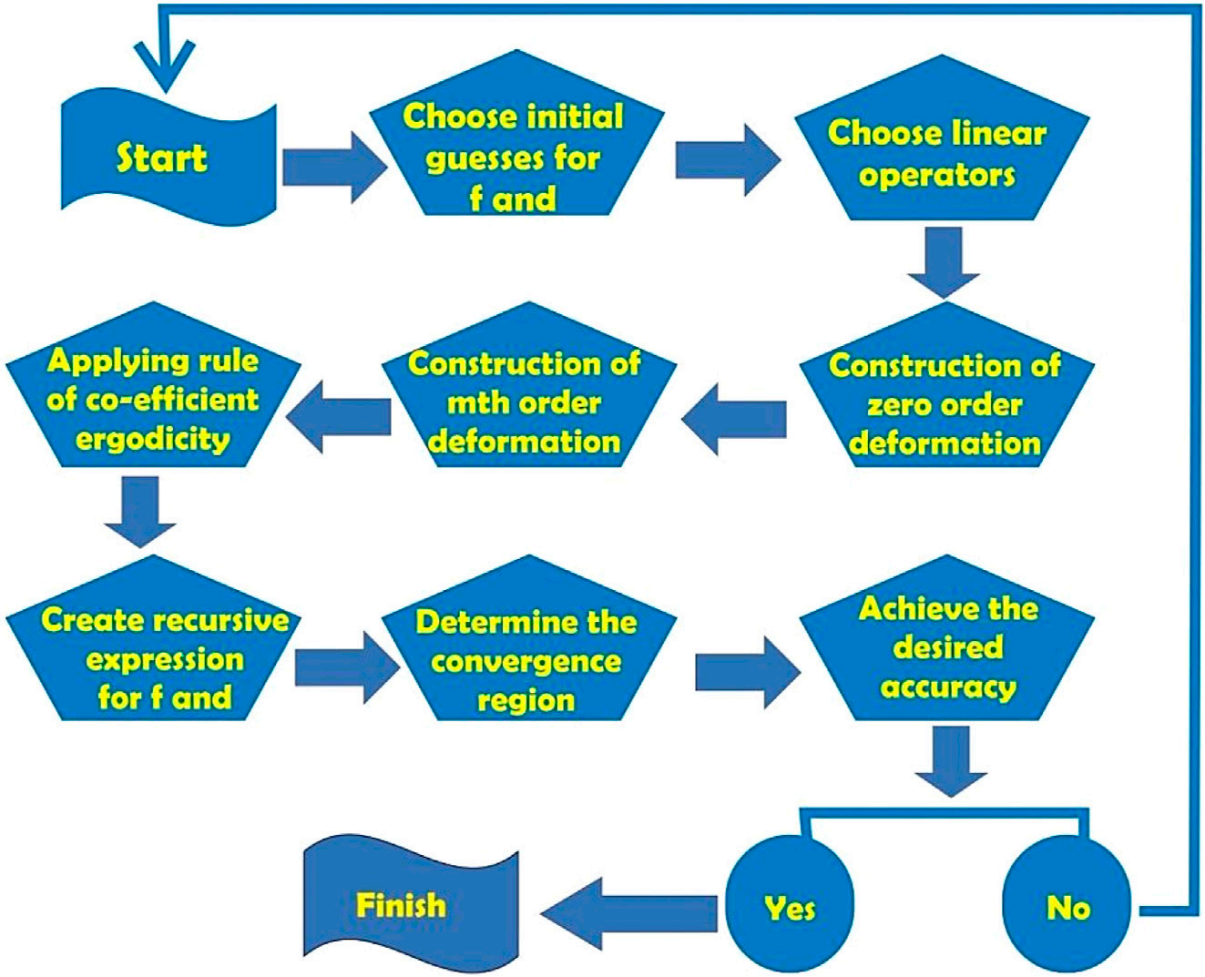
Flowchart of the HAM.

The homotopy analysis method (HAM) is applied to solve Equations 10–12, 13–15, and 16–18, subject to the boundary conditions given in Equation 19. The flowchart illustrating the HAM is shown in Figure 2.

The auxiliary parameter-containing solutions modify and regulate the rate of convergence of the solutions. The following Equation 32 represents how the primary guesses are chosen.

$$F_{0}\left(\eta \right)=1-x^{2},\theta_{0}\left(\eta \right)=1,\gamma_{0}\left(\eta \right)=1.$$ (32)

We consider the linear operators to be $L_{F},L_{\theta },\text{and }L_{\gamma }$ , which are defined in Equation 33 as follows:

$$L_{F}\left(F\right)=\frac{d^{3}F}{d\eta^{3}},L_{\theta }\left(\theta \right)=\frac{d^{2}\theta }{d\eta^{2}},L_{\gamma }\left(\gamma \right)=\frac{d^{2}\gamma }{d\eta^{2}}.$$ (33)

These operators possess the following characteristics, as given in Equation 34:

$$L_{F}\left(c_{1}+c_{2}\eta +c_{3}\eta^{2}\right)=0,L_{\theta }\left(c_{4}+c_{5}\eta \right)=0,L_{\gamma }\left(c_{6}+c_{7}\eta \right)=0,$$ (34)

where $c_{i}\left(i=1-7\right)$ are the constants in the general solution. The resultant non-linear operatives $N_{F},N_{\theta },{and\;N}_{\gamma }$ are given in Equations 35–37 as follows:

$$\begin{array}{l} N_{F}\left[F\left(\eta ;p\right)\right]=\frac{\partial^{3}F\left(\eta ;p\right)}{\partial \eta^{3}}+\left(1/\frac{\mu_{nf}}{\mu_{f}}\right)2\alpha R_{e}F\left(\eta ;p\right)\frac{\partial F\left(\eta ;p\right)}{\partial \eta }\\ -4\alpha^{2}R_{e}\frac{\partial F\left(\eta ;p\right)}{\partial \eta }-\left(\frac{\sigma_{nf}}{\sigma_{f}}/\frac{\mu_{nf}}{\mu_{f}}\right)M\frac{\partial F\left(\eta ;p\right)}{\partial \eta }, \end{array}$$ (35)

$$N_{\theta }\left[F\left(\eta ;p\right),\theta \left(\eta ;p\right)\right]=\left(\frac{k_{nf}}{k_{f}}+Rd\right)\frac{\partial^{2}\theta \left(\eta ;p\right)}{\partial \eta^{2}}+\frac{\sigma_{nf}}{\sigma_{f}}\alpha \mathrm{Pr}EcM{\left(F\left(\eta ;p\right)\right)}^{2},$$ (36)

$$N_{\gamma }\left[\gamma \left(\eta ;p\right)\right]=\frac{\partial^{2}\gamma \left(\eta ;p\right)}{\partial \eta^{2}}+ScK_{1}\gamma \left(\eta ;p\right).$$ (37)

The zeroth-order problems from Equations 10–12, 13–15, and 16–18 provide an overview of the fundamental concepts of the HAM, as presented in Equations 38–40:

$$\left(1-p\right)L_{F}\left[F\left(\eta ;p\right)-F_{0}\left(\eta \right)\right]=p\hbar_{F}N_{F}\left[F\left(\eta ;p\right)\right],$$ (38)

$$\left(1-p\right)L_{\theta }\left[\theta \left(\eta ;p\right)-\theta_{0}\left(\eta \right)\right]=p\hbar_{\theta }N_{\theta }\left[F\left(\eta ;p\right),\theta \left(\eta ;p\right)\right],$$ (39)

$$\left(1-p\right)L_{\gamma }\left[\gamma \left(\eta ;p\right)-\gamma_{0}\left(\eta \right)\right]=p\hbar_{\gamma }N_{\gamma }\left[\gamma \left(\eta ;p\right)\right].$$ (40)

The equivalent boundary conditions are given in Equation 41 as follows:

$$\begin{array}{l} {\left.F\left(\eta ;p\right)\right|}_{\eta =0}=1,{\left.\frac{dF\left(\eta ;p\right)}{d\eta }\right|}_{\eta =0}=0,{\left.\frac{d\theta \left(\eta ;p\right)}{d\eta }\right|}_{\eta =0}=0,{\left.\frac{d\gamma \left(\eta ;p\right)}{d\eta }\right|}_{\eta =0}=0,\\ {\left.F\left(\eta ;p\right)\right|}_{\eta =1}=0,{\left.\theta \left(\eta ;p\right)\right|}_{\eta =1}=1,{\left.\gamma \left(\eta ;p\right)\right|}_{\eta =1}=1. \end{array}$$ (41)

The incorporating parameter is denoted by $p\in \left[0,1\right]$ , and $\hbar_{F},\hbar_{\theta },\hbar_{\gamma }$ are used to regulate the solution’s convergence. When p=0 and p=1 , we obtain Equation 42 as follows:

$$F\left(\eta ;1\right)=F\left(\eta \right),\theta \left(\eta ;1\right)=\theta \left(\eta \right),\gamma \left(\eta ;1\right)=\gamma \left(\eta \right)$$ (42)

The Taylor’s series expansion of $F\left(\eta ;p\right),\theta \left(\eta ;p\right)\;\gamma \left(\eta ;p\right)$ about p = 0 is given in Equation 43 as follows:

$$\begin{array}{l} F\left(\eta ;p\right)=F_{0}\left(\eta \right)+\sum_{m=1}^{\infty }F_{m}\left(\eta \right)p^{m},\theta \left(\eta ;p\right)=\theta_{0}\left(\eta \right)+\sum_{m=1}^{\infty }\theta_{m}\left(\eta \right)p^{m},\\ V\left(\eta ;p\right)=\gamma_{0}\left(\eta \right)+\sum_{m=1}^{\infty }\gamma_{m}\left(\eta \right)p^{m}, \end{array}$$ (43)

where Equation 44 presents the following:

$$F_{m}\left(\eta \right)={\left.\frac{1}{m!}\frac{\partial F\left(\eta ;p\right)}{\partial \eta }\right|}_{p=0},\theta_{m}\left(\eta \right)={\left.\frac{1}{m!}\frac{\partial \theta \left(\eta ;p\right)}{\partial \eta }\right|}_{p=0},\gamma_{m}\left(\eta \right)={\left.\frac{1}{m!}\frac{\partial \gamma \left(\eta ;p\right)}{\partial \eta }\right|}_{p=0}.$$ (44)

In order to ensure that Equation 31 converges at p = 1, the secondary constraints $\hbar_{F},\hbar_{\theta },{\text{and}\;\hbar }_{\gamma }$ are selected. Substituting p=1 in Equation 31, we acquire Equation 45 as follows:

$$F\left(\eta \right)=F_{0}\left(\eta \right)+\sum_{m=1}^{\infty }F_{m}\left(\eta \right),\theta \left(\eta \right)=\theta_{0}\left(\eta \right)+\sum_{m=1}^{\infty }\theta_{m}\left(\eta \right),\gamma \left(\eta \right)=\gamma_{0}\left(\eta \right)+\sum_{m=1}^{\infty }\gamma_{m}\left(\eta \right)$$ (45)

The following Equation 46 is satisfied by the $m^{th}$ -order problem:

$$\begin{array}{l} L_{F}\left[F_{m}\left(\eta \right)-\omega_{m}F_{m-1}\left(\eta \right)\right]=\hbar_{F}R_{m}^{F}\left(\eta \right),L_{\theta }\left[\theta_{m}\left(\eta \right)-\omega_{m}\theta_{m-1}\left(\eta \right)\right]=\hbar_{\theta }R_{m}^{\theta }\left(\eta \right),\\ L_{\gamma }\left[\gamma_{m}\left(\eta \right)-\omega_{m}\gamma_{m-1}\left(\eta \right)\right]=\hbar_{\gamma }R_{m}^{\gamma }\left(\eta \right). \end{array}$$ (46)

The corresponding boundary conditions are given in Equation 47 as follows:

$$\begin{array}{l} f_{m}\left(0\right)=f_{m}^{\prime }\left(0\right)=\theta_{m}^{\prime }\left(0\right)=\gamma_{m}^{\prime }\left(0\right)=0\\ f_{m}\left(1\right)=\theta_{m}\left(1\right)=\gamma_{m}\left(1\right)=0 \end{array}$$ (47)

Here, Equations 48–50 present the following:

$$R_{m}^{f}\left(\eta \right)=F_{m-1}^{\prime \prime \prime }+\left(1/\frac{\mu_{nf}}{\mu_{f}}\right)2\alpha R_{e}\sum_{k=0}^{m-1}F_{m-1-k}F_{k}^{\prime }-4\alpha^{2}R_{e}F_{m-1}^{\prime }-\left(\frac{\sigma_{nf}}{\sigma_{f}}/\frac{\mu_{nf}}{\mu_{f}}\right)MF_{m-1}^{\prime },$$ (48)

$$R_{m}^{\theta }\left(\eta \right)=\left(\frac{k_{nf}}{k_{f}}+Rd\right)\theta_{m-1}^{\prime \prime }+\frac{\sigma_{nf}}{\sigma_{f}}\alpha \mathrm{Pr}EcM\sum_{k=0}^{m-1}F_{m-1-k}^{\prime }F_{k}^{\prime },$$ (49)

$$R_{m}^{\gamma }\left(\eta \right)=\gamma_{m-1}^{\prime \prime }+ScK_{1}\gamma_{m-1},$$ (50)

where

$$\omega_{m}=\left\{\begin{array}{l} 0,\text{if }p\leq 1\\ 1,\text{if }p>1 \end{array}\right..$$

## 7 Result and discussion on physical parameters

Semi-analytical solutions were found for the converted radial momentum, energy, and concentration Equations 2, 4, 5, according to the boundary constraints Equations 6, 7 using HAM in Mathematica. The effects of a variety of factors on velocity and temperature profiles for Al_2_O_3_–H_2_O, γ -Al_2_O_3_–H_2_O, and Al_2_O_3_–EG are examined, including nanoparticle volume fraction φ, the magnetic parameter M, the radiation parameter Rd, and the Eckert number Ec in convergent/divergent channels. We examined the effects of governing parameter values, such as chemical reactions and the Schmidt number, on the concentration profiles for Al_2_O_3_–H_2_O, γ -Al_2_O_3_–H_2_O, and γ-Al_2_O_3_–C_2_H_6_O_2_ nanofluids. Table 1 provides an overview of the thermo-physical characteristics of the nanofluids. Table 2 exhibits the behavior of skin friction for various parameters. Table 3 shows the impact of α and Ec. Table 4 provides variation in the Sherwood number for K_1_ and Sc.

### 7.1 Velocity field

**FIGURE 3:**
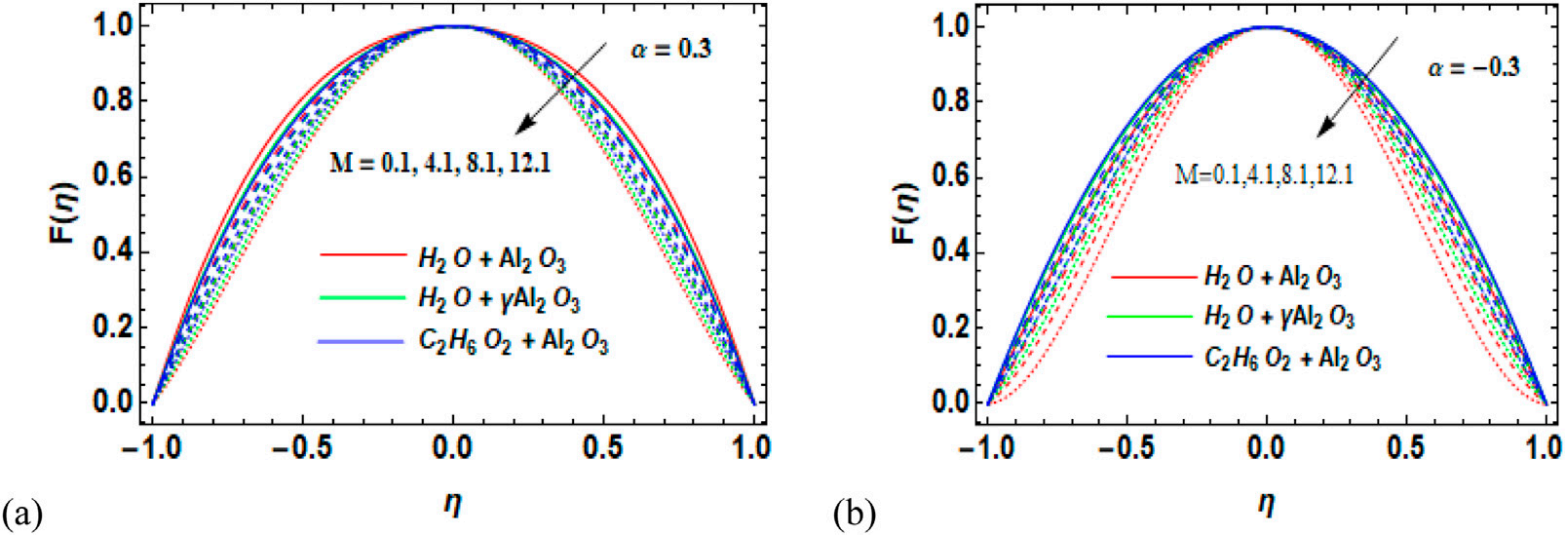
**(a, b)** Variation in the magnetic parameter (M) against velocity (F $\left(\eta \right)$ ) for **(a)** the divergent channel and **(b)** the convergent channel for Al_2_O_3_–H_2_O, γ -Al_2_O_3_–H_2_O, and γ-Al_2_O_3_–C_2_H_6_O_2_.

**FIGURE 4:**
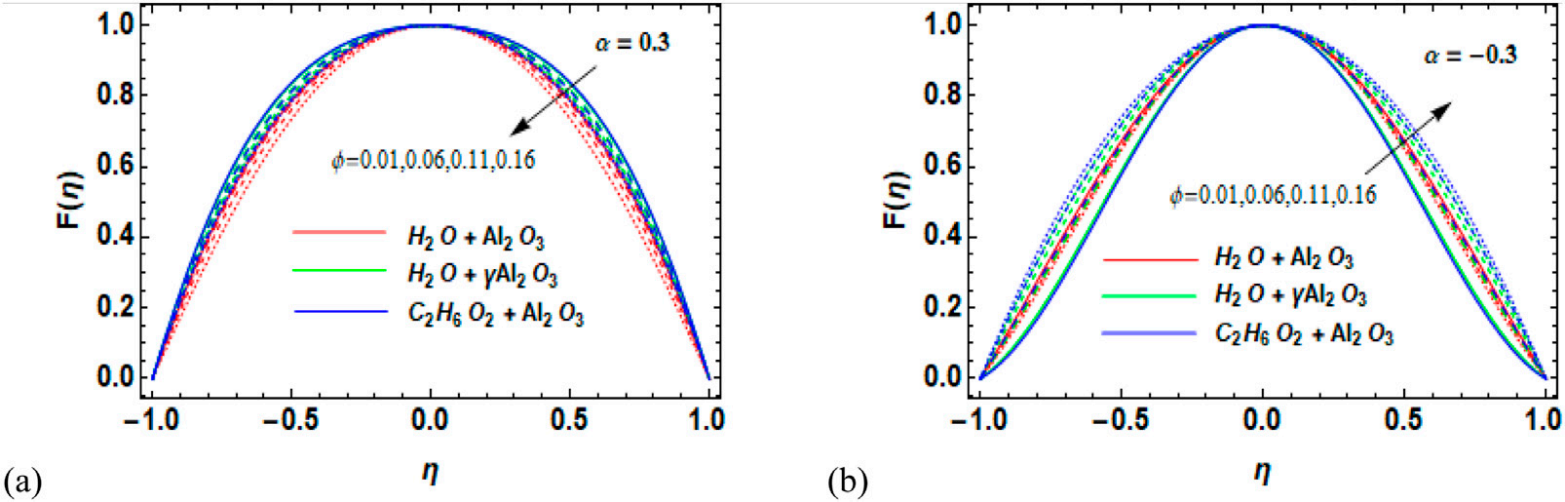
**(a, b)** Variation in the volume fraction (*ϕ*) against velocity (F $\left(\eta \right)$ ) for **(a)** the divergent channel and **(b)** the convergent channel for Al_2_O_3_–H_2_O, γ -Al_2_O_3_–H_2_O, and γ-Al_2_O_3_–C_2_H_6_O_2_.

**FIGURE 5:**
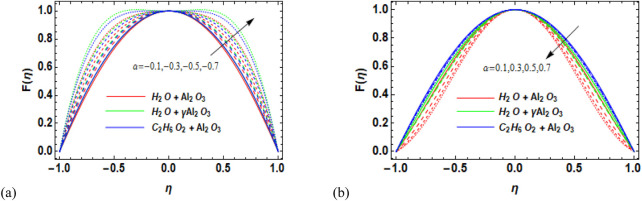
**(a, b)** Variation in **(a)** the diverging angle ( $\left.\alpha >0\right)$ and **(b)** the convergent angle ( $\left.\alpha <0\right)$ against velocity (F $\left(\eta \right)$ ) for Al_2_O_3_–H_2_O, γ -Al_2_O_3_–H_2_O, and γ-Al_2_O_3_–C_2_H_6_O_2_.

Figures 3a, b display the trend of a magnetic parameter with respect to the velocity $F\left(\eta \right)$ of the nanofluid for Al_2_O_3_–H_2_O, γ -Al_2_O_3_–H_2_O, and γ-Al_2_O_3_–C_2_H_6_O_2_ in divergent/convergent channels, respectively. The magnetic parameter plays an important role in the flow performance of nanofluids. Here are several reasons why the magnetic parameter is important in the nanofluid flow. It affects the velocity, pressure circulation, and heat transmission characteristics of the nanofluid. We can use the magnetic parameter to control and manipulate the flow performance of nanofluids. It is augmented by the heat transfer of nanofluids. It also affects the rheological properties of nanofluids. It is used in biomedical applications for targeted drug delivery, hyperthermia therapy, and magnetic resonance imaging (MRI). The fluid flow is slowed by Lorentz forces, which are created by larger physical changes in the magnetic parameter. The overall nanofluid velocity decreases with the increasing magnetic parameter M for both divergent and convergent channels. Figure 3a indicates a fast decrease in the velocity of Al_2_O_3_–H_2_O, followed by γ -Al_2_O_3_–H_2_O and γ-Al_2_O_3_–C_2_H_6_O_2_, respectively. The impact of the solid volume fraction ( ϕ ) on the velocity of the fluid is shown in Figures 4a, b. The solid volume fraction ( ϕ ) is a precarious parameter that ominously influences the nanofluid flow behavior, thermal assets, stability, and act of nanofluids. Careful management and optimization are needed for comprehending the full potential of nanofluid-based technologies in several engineering, biomedical, and environmental applications. Physically, adding nanoparticles to the pure fluid causes the fluid’s density to increase, which makes it harder for the fluid to pass through the channel. Figure 4a shows that an increase in the volume fraction of solids in the diverging channel results in a reduction in the fluid velocity. Figure 4b, on the other hand, shows a reverse pattern for the convergent channel, i.e., as the solid volume percentage increases, so does the velocity. The impact of diverging/converging channels on the velocity profile of nanofluids is exhibited in Figures 5a, b, respectively. Figure 5a shows that variation in the diverging channel ( α>0 ) causes a decrease in the velocity of the nanofluids. Moreover, a greater decrease in velocity was observed for Al_2_O_3_–H_2_O, γ -Al_2_O_3_–H_2_O, and γ-Al_2_O_3_–C_2_H_6_O_2_. On the other hand, the velocity increases with variations in the converging channel $\left(\alpha <0\right)$ , as shown in Figure 5b.

### 7.2 Temperature field

**FIGURE 6:**
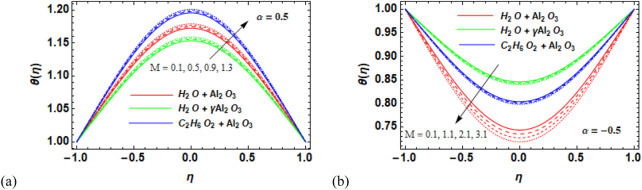
**(a, b)** Variation in the magnetic parameter (M) against the temperature profile $\theta \left(\eta \right)$ for the divergent channel $\left(\alpha >0\right)$ for Al_2_O_3_–H_2_O, γ Al_2_O_3_–H_2_O, and γ Al_2_O_3_–C_2_H_6_O_2_.

**FIGURE 7:**
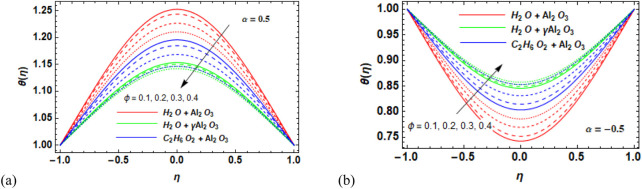
**(a, b)** Variation in the volume fraction (*ϕ*) against the temperature profile $\theta \left(\eta \right)$ for **(a)** the divergent channel and **(b)** the convergent channel for Al_2_O_3_–H_2_O, γ -Al_2_O_3_–H_2_O, and γ-Al_2_O_3_–C_2_H_6_O_2_.

**FIGURE 8:**
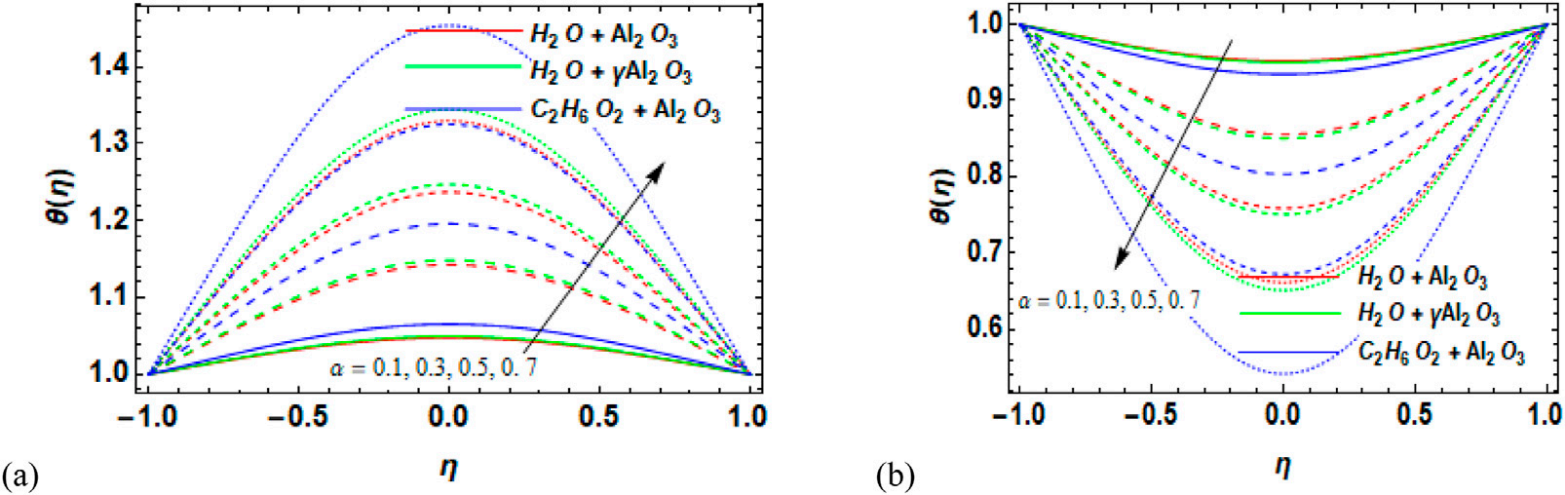
**(a,b)** Variation in **(a)** the diverging angle ( $\left.\alpha >0\right)$ and **(b)** the converging angle ( $\left.\alpha <0\right)$ against the temperature profile $\theta \left(\eta \right)$ for Al_2_O_3_–H_2_O, γ -Al_2_O_3_–H_2_O, and γ-Al_2_O_3_–C_2_H_6_O_2_.

**FIGURE 9:**
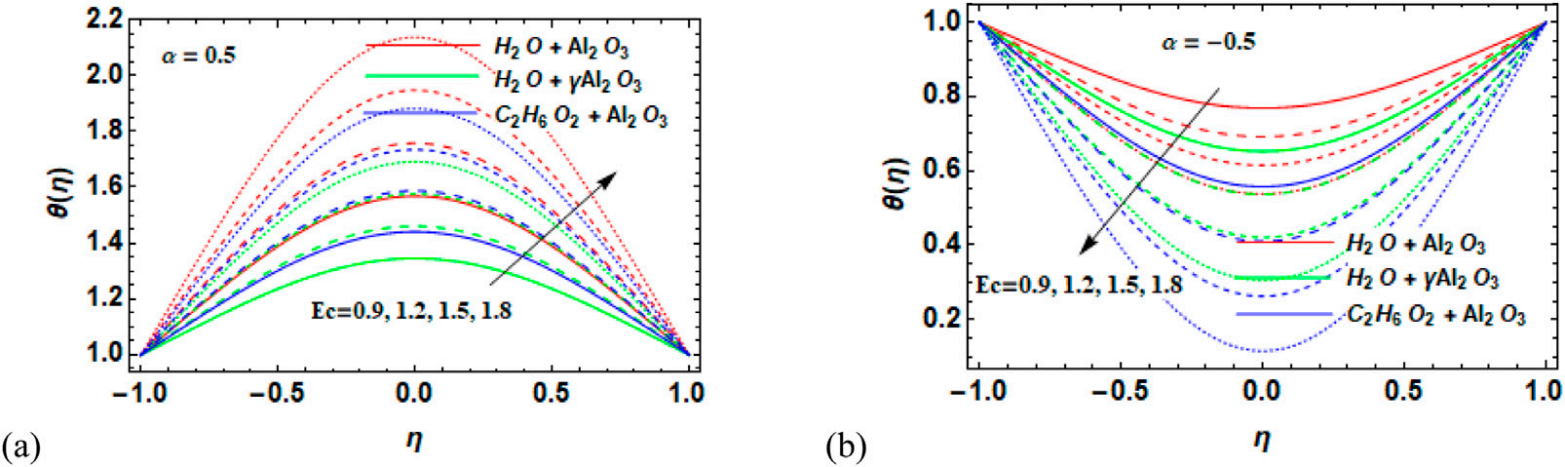
**(a, b)** Variation in the Eckert number (Ec) against the temperature profile $\theta \left(\eta \right)$ for **(a)** the divergent channel and **(b)** the convergent channel for Al_2_O_3_–H_2_O, γ -Al_2_O_3_–H_2_O, and γ-Al_2_O_3_–C_2_H_6_O_2_.

**FIGURE 10:**
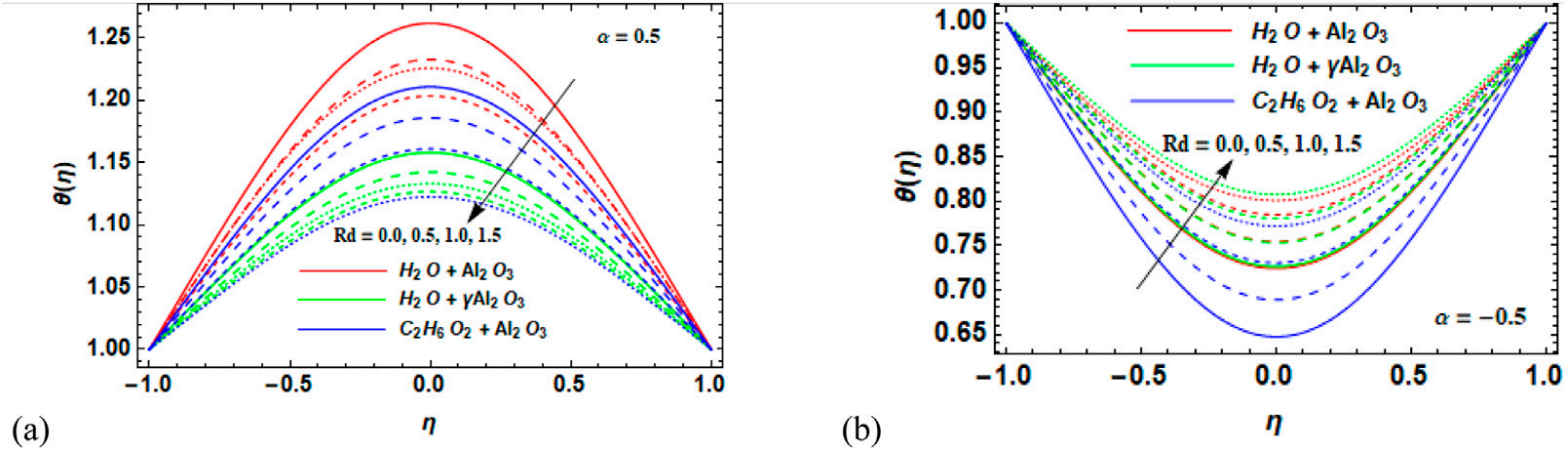
**(a, b)** Variation in the radiation parameter (Rd) against the temperature profile $\theta \left(\eta \right)$ for **(a)** the divergent channel and **(b)** the convergent channel for Al_2_O_3_–H_2_O, γ -Al_2_O_3_–H_2_O, and γ-Al_2_O_3_–C_2_H_6_O_2_.

Over the desired domain, the temperature of the nanofluids was strongly influenced by the flow parameters. Consequently, the behavior of the temperature θ(*η*) versus the magnetic number M for Al_2_O_3_–H_2_O, γ -Al_2_O_3_–H_2_O, and γ-Al_2_O_3_–C_2_H_6_O_2_ was depicted in Figures 6a, b. A careful analysis of the data revealed that the used nanofluids’ temperature increases with an increas in the magnetic field strength. Through the physical imposition of a dissipation function in the model, energy is transferred from high-temperature particles to lower-temperature particles by improving the fluid’s internal energy. As a result, the temperature of the nanofluids increases overall. There is a noticeable increase in γAl_2_O_3_–C_2_H_6_O_2_ and Al_2_O_3_–H_2_O compared to γ -Al_2_O_3_–H_2_O. The impact of the solid volume fraction (φ) on the temperature profile of fluid through divergent and convergent channels is shown in Figures 7a, b, respectively. Figure 7a suggests that an increase in the solid volume fraction in the diverging channels leads to a decrease in temperature distribution. Moreover, there is a sharp decrease in temperature for Al_2_O_3_–H_2_O, followed by γ -Al_2_O_3_–H_2_O. However, the least decrease in temperature is observed for γ-Al_2_O_3_–C_2_H_6_O_2_. It is evident from Figure 7b that the temperature distribution is favored by an increase in the solid volume fraction in the convergent channel. However, there is least augmentation in temperature observed for γ -Al_2_O_3_–H_2_O. The influence of divergent and convergent channels on the fluid temperature distribution is depicted in Figures 8a, b, respectively. The plotted result analysis shows that temperature increases for more diverging walls (Figure 8a) and decreases for convergent walls (Figure 8b). The temperature increases significantly in γ-Al_2_O_3_–C_2_H_6_O_2_ and γ -Al_2_O_3_–H_2_O compared to Al_2_O_3_–H_2_O for divergent walls. Similarly, temperature considerably decreases in γ-Al_2_O_3_–C_2_H_6_O_2_ and γ -Al_2_O_3_–H_2_O compared to Al_2_O_3_–H_2_O for convergent walls. Figures 9a, b illustrate how variations in the Eckert number affect the temperature distribution in diverging and converging channels. The Eckert number plays a vital role in influencing numerous features of nanofluid flow behavior, along with heat transfer and thermal performance. It is very important in terms of kinetic and thermal energy in the fluid flow, permitting engineers and researchers to design and enhance nanofluid systems for several real-world applications. A careful examination of the data revealed that the used nanofluids’ temperature increases by strengthening their Eckert numbers. The model’s imposed dissipation function physically increases the fluid’s internal energy, facilitating the movement of energy from particles with higher temperatures to those with lower temperatures. As a result, the temperature of the nanofluids increases overall. The temperature increases noticeably in Al_2_O_3_–H_2_O, followed by γ-Al_2_O_3_–C_2_H_6_O_2_ and γ -Al_2_O_3_–H_2_O, respectively. The effect of increasing Rd on the temperature profile is shown in Figures 10a, b. The graph shows that as heat radiation is steadily increased, the temperature profile decreases for the divergent channel (Figure 10a). It is due to the fact that the presence of the radiation parameter leads to thinning of the thermal layer. However, for the convergent channel (Figure 10b), temperature increases for the increase in the radiation parameter.

### 7.3 Concentration field

**FIGURE 11:**
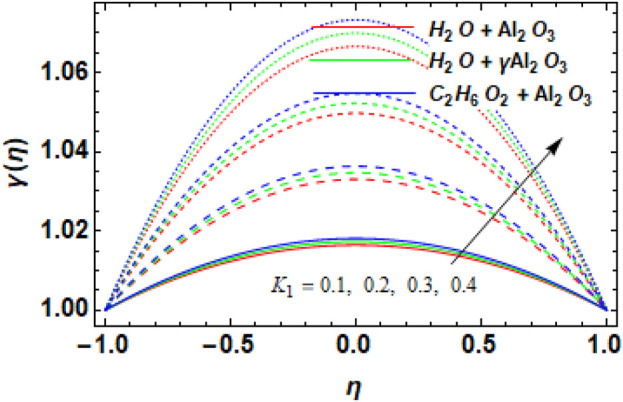
Variation in chemical reaction parameter $\left(K_{1}\right)$ versus concentration profile $\left(\gamma \left(\eta \right)\right)$ .

**FIGURE 12:**
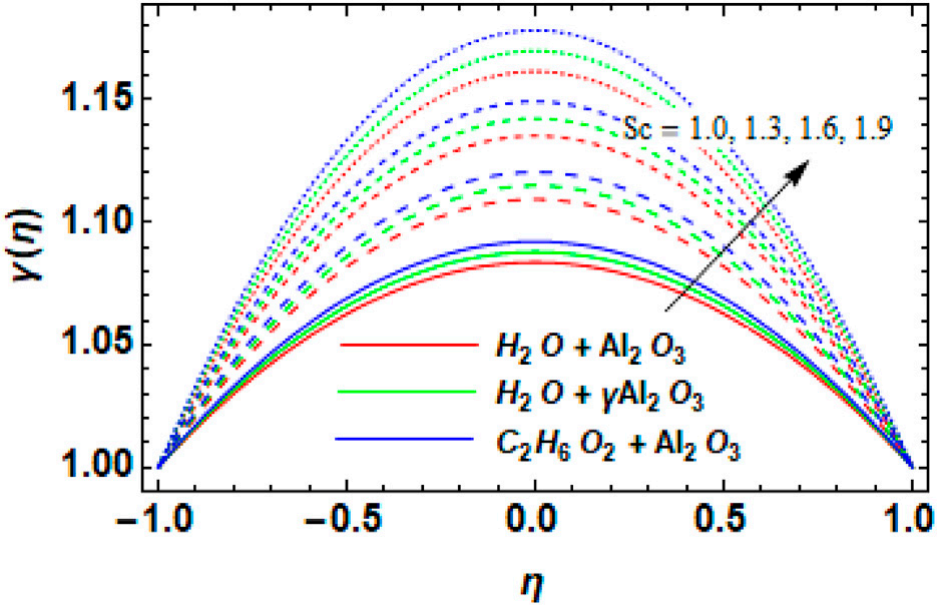
Variation in the Schmidt number $\left(Sc\right)$ versus concentration profile $\left(\gamma \left(\eta \right)\right)$ .

Figure 11 shows the impact of the increasing $\left(K_{1}\right)$ on the concentration profile $\left(\gamma \left(\eta \right)\right)$ . The figure reveals that increasing the value of the chemical reaction parameter results in increasing the concentration profile. Moreover, there is a significant increase observed in the concentration profiles of γ-Al_2_O_3_–C_2_H_6_O_2_, followed by γ -Al_2_O_3_–H_2_O and Al_2_O_3_–H_2_O, respectively. The reason for this is that in this case, an increase in the chemical reaction parameter causes the rate of reaction to increase. The faster conversion of reactants into products as a result of the higher reaction rate has an impact on the nanofluid concentration. Higher reaction rates result in higher concentrations of nanoparticles because the reaction parameter affects the production or consumption of nanoparticles in the nanofluid. The behavior of increasing Sc versus the concentration profile is exhibited in Figure 12. It is evident from the figure that, overall, all used nanofluids exhibit an enhanced concentration profile with increasing values of the Schmidt number.

### 7.4 Discussion on the graphical and numerical analysis of physical quantities

Table 5 exhibits the trend of skin fiction for increasing values of α , $R_{e}$ , and M for ${Al}_{2}O_{3}-H_{2}O$ , ${Al}_{2}O_{3}-C_{2}H_{6}O_{2}$ , $\gamma {Al}_{2}O_{3}-H_{2}O$ , and $\gamma {Al}_{2}O_{3}-C_{2}H_{6}O_{2}$ . The table shows that skin friction increases with increasing values of α for all nanofluids while keeping the values of $R_{e}$ and M constant. A similar trend is observed in skin friction in all used nanofluids for increasing values of $R_{e}$ and M. Table 6 shows the behavior of the Nusselt number Nu for the increasing values of α and the Eckert number Ec. Table 6 shows that as the values of α and Ec are increased separately, the Nusselt number increases for all nanofluids used. This indicates that the nanofluids have faster heat transfer ability. Table 7 indicates the trend of the Sherwood number for K_1_ and Sc. A rapid increase in the Sherwood number is observed for increasing values of K_1_ by fixing Sc for all used nanofluids. Similarly, a significant enhancement in the Sherwood number is also observed in all nanofluids for increasing values of Sc when the value of K_1_ is kept constant.

**TABLE 5:** Variation in skin friction in nanofluids for $R_{e}$ and M.

α	$R_{e}$	M	${Al}_{2}O_{3}-H_{2}O$ ( $C_{f}$ )	${Al}_{2}O_{3}-C_{2}H_{6}O_{2}$ ( $C_{f}$ )	$\gamma {Al}_{2}O_{3}-H_{2}O\;\left(C_{f}\right)$	$\gamma {Al}_{2}O_{3}-C_{2}H_{6}O_{2}\left(C_{f}\right)$
0.1	0.3	0.3	1.9960	1.5446	2.1120	1.5493
0.2			1.9970	1.5450	2.1131	1.5497
0.3			1.9995	1.5467	2.1160	1.5514
0.4			2.0037	1.5496	2.1205	1.5544
0.1	0.3		1.9960	1.5446	2.1120	1.5493
	0.4		1.9955	1.5441	2.1115	1.5488
	0.5		1.9950	1.5437	2.1111	1.5484
	0.6		1.9946	1.5432	2.1106	1.5479
	0.3	0.3	1.9960	1.5446	2.1120	1.5493
		0.4	1.9977	1.5459	2.1136	1.5505
		0.5	1.9994	1.5472	2.1152	1.5518
		0.6	2.0011	1.5485	2.1169	1.5531

**TABLE 6:** Variation in the Nusselt Number Nu for α and Eckert number Ec.

α	Ec	${Al}_{2}O_{3}-H_{2}O$ ( Nu )	${Al}_{2}O_{3}-C_{2}H_{6}O_{2}$ ( Nu )	$\gamma {Al}_{2}O_{3}-H_{2}O\;\left(Nu\right)$	$\gamma {Al}_{2}O_{3}-C_{2}H_{6}O_{2}\left(Nu\right)$
0.1	0.3	1.1663	1.1675	1.0369	1.0975
0.2		1.2244	1.2256	1.0888	1.1523
0.3		1.3190	1.3204	1.1733	1.2415
0.4		1.4504	1.4519	1.2903	1.3652
0.1		1.1663	1.1675	1.0369	1.0975
		0.94265	0.94363	0.83814	0.88706
		0.80842	0.80927	0 .71884	0.76078
		0.71894	0.71970	0.63931	0.67659
	0.3	1.1663	1.1675	1.0369	1.0975
	0.4	1.5551	1.5567	1.3826	1.4633
	0.5	1.9439	1.9459	1.7282	1.8292
	0.6	2.3327	2.3351	2.0739	2.1951

**TABLE 7:** Variation in the Sherwood number for the chemical reaction parameter (K1) and Schmidt number (Sc).

K1	Sc	${Al}_{2}O_{3}-H_{2}O$ ( Shx )	${Al}_{2}O_{3}-C_{2}H_{6}O_{2}$ ( Shx )	$\gamma {Al}_{2}O_{3}-H_{2}O\left(Shx\right)$	$\gamma {Al}_{2}O_{3}-C_{2}H_{6}O_{2}\left(Shx\right)$
0.6	0.6	0.18748	0.18748	0.18748	0.18748
0.7		0.21949	0.21949	0.21949	0.21949
0.8		0.25171	0.25171	0.25171	0.25171
0.9		0.28414	0.28414	0.28414	0.28414
0.6	0.6	0.18748	0.18748	0.18748	0.18748
	0.7	0.21949	0.21949	0.21949	0.21949
	0.8	0.25171	0.25171	0.25171	0.25171
	0.9	0.28414	0.28414	0.28414	0.28414

**FIGURE 13:**
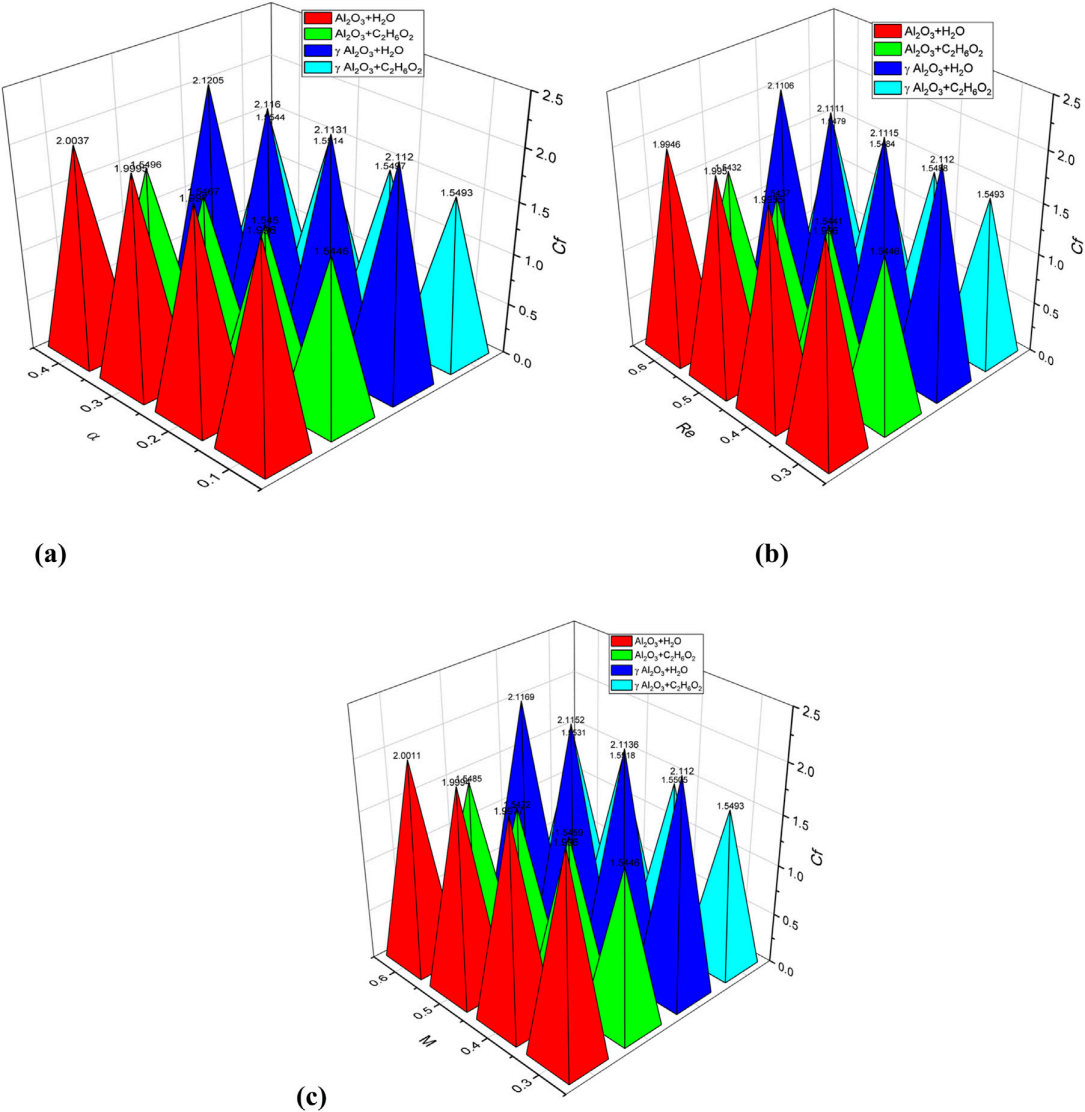
**(a–c)** Skin friction (Cf) versus convergent/divergent parameter α , Renolds number (Re), and magnetic parameter (M).

**FIGURE 14:**
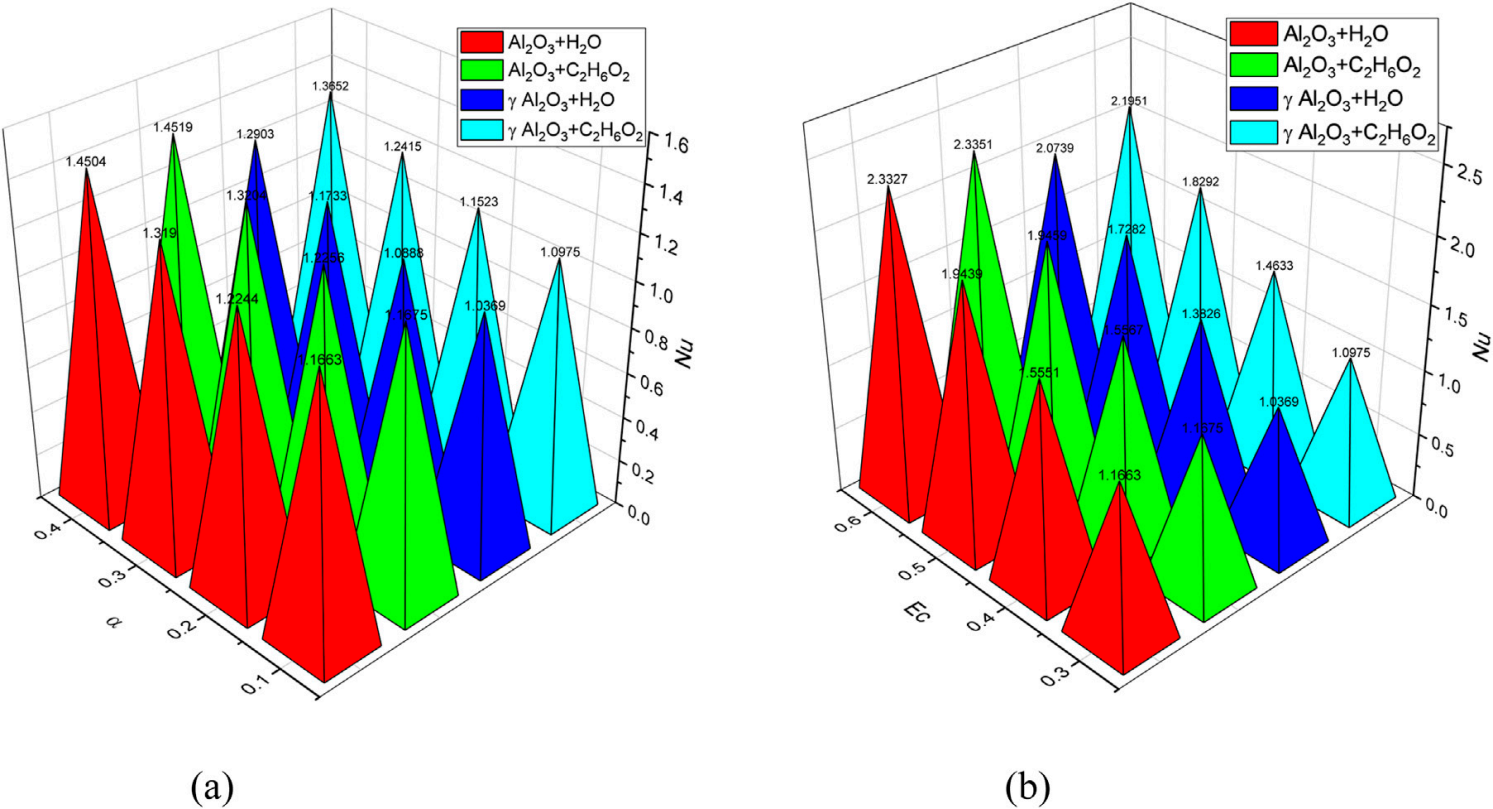
**(a–b)** Behavior of the Nusselt number for the variation in α and the Eckert number (Ec).

**FIGURE 15:**
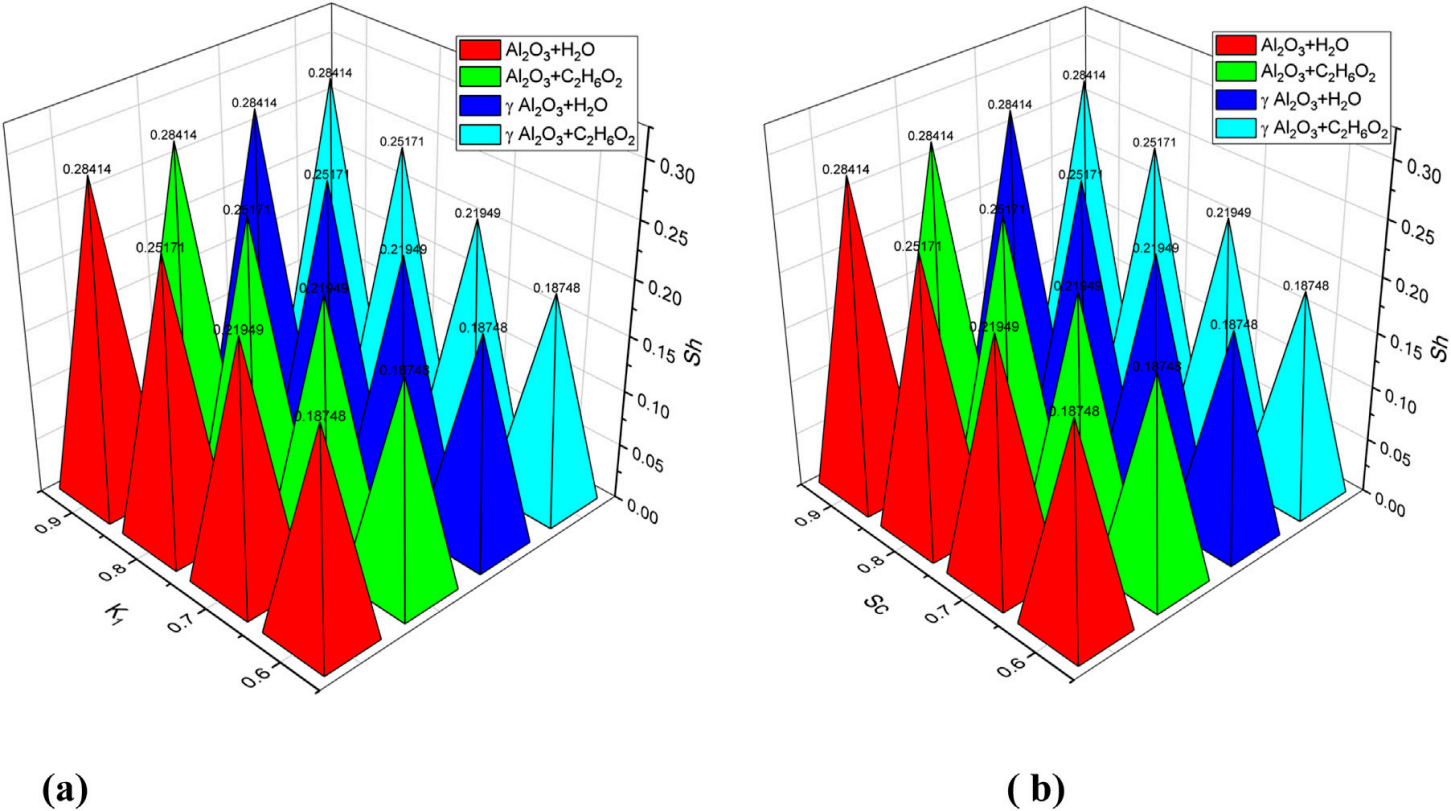
**(a–b)** Behavior of the Sherwood number (S_h_) for the variation in the chemical reaction parameter ( $\left.K_{1}\right)$ and Schmidt number S_c_.

**FIGURE 16:**
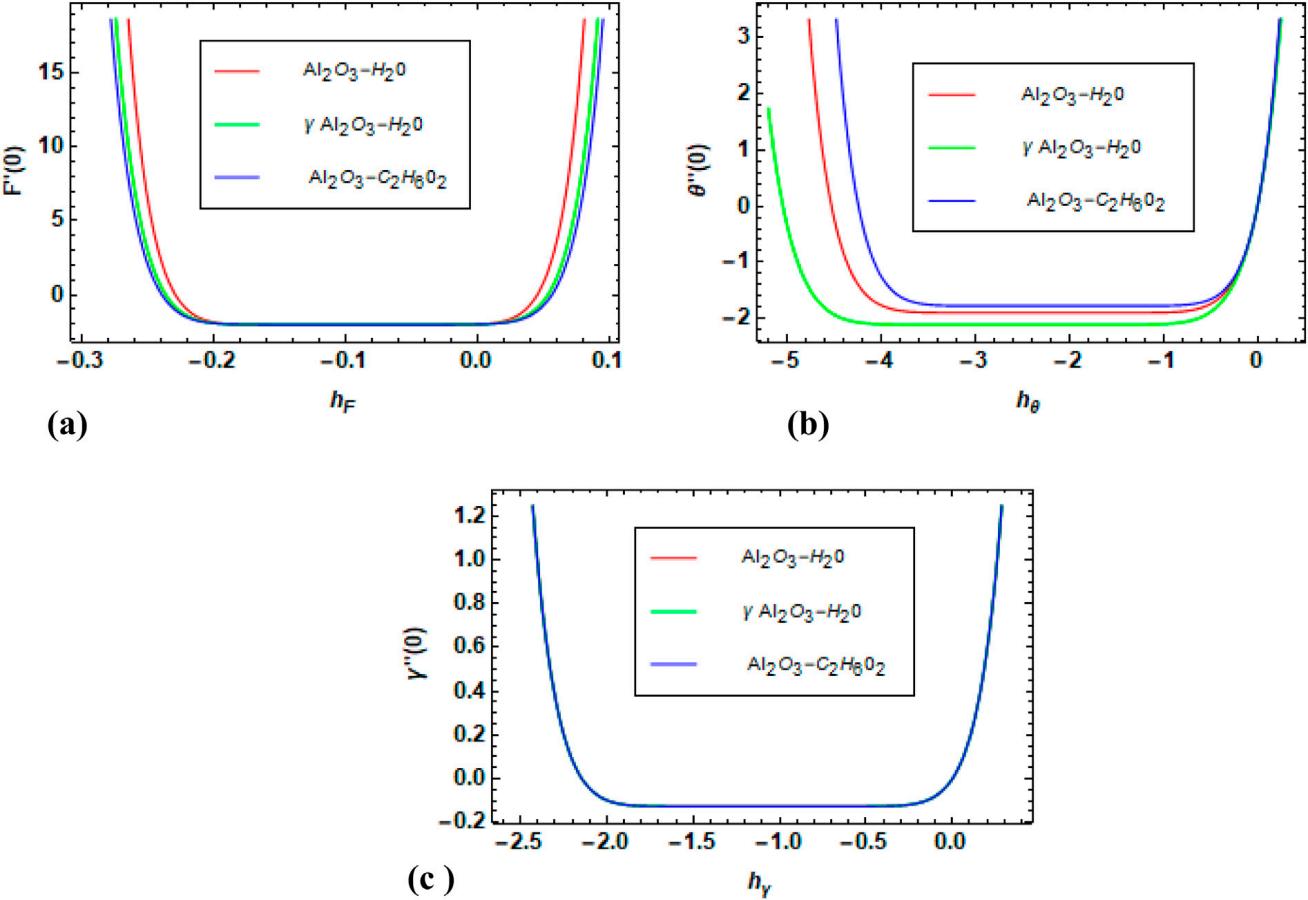
**(a–c)** h-curves of (F $\left(\eta \right)$ ), $\theta \left(\eta \right)$ , and $\left(\gamma \left(\eta \right)\right)$ for Al_2_O_3_–H_2_O, γ -Al_2_O_3_–H_2_O, and γ-Al_2_O_3_–C_2_H_6_O_2._

Figure 13a exhibits the trend of C_f_ for increasing values of α. An overall enhancement in skin friction is observed for increasing values of α for nanofluids ${Al}_{2}O_{3}-H_{2}O$ , ${Al}_{2}O_{3}-C_{2}H_{6}O_{2}$ , $\gamma {Al}_{2}O_{3}-H_{2}O$ , and $\gamma {Al}_{2}O_{3}-C_{2}H_{6}O_{2}$ . It is further observed that more enhancement is observed in skin friction for $\gamma {Al}_{2}O_{3}-H_{2}O$ , followed by ${Al}_{2}O_{3}-H_{2}O$ and $\gamma {Al}_{2}O_{3}-C_{2}H_{6}O_{2}$ , respectively. Figure 13b shows the evolution of C_f_ for increasing values of the Reynolds number (Re). Skin friction for used nanofluids is decreased for increasing values of the Reynolds number. Figure 13c shows an increased behavior of Cf for increasing values of the magnetic parameter (M) for all used nanofluids, i. e., $\gamma {Al}_{2}O_{3}-H_{2}O$ , ${Al}_{2}O_{3}-H_{2}O$ , and $\gamma {Al}_{2}O_{3}-C_{2}H_{6}O_{2}$ . Figure 14a demonstrates the trend of Nu for increasing values of α. It is observed from the figure that generally Nu has an increasing trend for increasing values of α for $\gamma {Al}_{2}O_{3}-H_{2}O$ , ${Al}_{2}O_{3}-H_{2}O$ , and $\gamma {Al}_{2}O_{3}-C_{2}H_{6}O_{2}$ nanofluids. This is due to the fact that the cross-sectional area increases and that the fluid velocity decreases with a greater angle of divergence. Because there is more surface area available for heat exchange, heat transmission is improved. As a result, as the angle of divergence increases, the Nusselt number also increases. In addition, ${Al}_{2}O_{3}-H_{2}O$ shows more increasing behavior, followed by $\gamma {Al}_{2}O_{3}-C_{2}H_{6}O_{2}$ and $\gamma {Al}_{2}O_{3}-H_{2}O$ , respectively. Variations in Nu for increasing values of Ec are depicted in Figure 14b. A sharp enhancement is observed in the Nusselt number of $\gamma {Al}_{2}O_{3}-H_{2}O$ , ${Al}_{2}O_{3}-H_{2}O$ , and $\gamma {Al}_{2}O_{3}-C_{2}H_{6}O_{2}$ nanofluids by increasing the Eckert number. This indicates that the mentioned nanofluids have a higher rate of heat transfer. Moreover, the graph also shows that the used nanofluids exhibit more or less a similar behavior in their enhancement of the Nusselt number. Figure 15a depicts the trend of Sh for increased values of $K_{1}$ . A similar and minor enhancement is observed in Sh for all used nanofluids, i.e., $\gamma {Al}_{2}O_{3}-H_{2}O$ , ${Al}_{2}O_{3}-H_{2}O$ , and $\gamma {Al}_{2}O_{3}-C_{2}H_{6}O_{2}$ when $K_{1}$ is increased. Figure 15b depicts the variation in Sh for increased values of Sc. Overall, there is an increased trend observed in Sh for increased values of Sc for used nanofluids. Figures 16a–c show the h-curve graphs of (F $\left(\eta \right)$ ), $\theta \left(\eta \right)$ , and $\left(\gamma \left(\eta \right)\right)$ for Al_2_O_3_–H_2_O, γ -Al_2_O_3_–H_2_O, and γ-Al_2_O_3_–C_2_H_6_O_2_.

### 7.5 Validation of the results

Adequate comparison and discussion on the results with the existing literature for $F^{\prime \prime }\left(0\right)$ , $\alpha =5^{0},\phi =0$ with different values of R_e_ are presented. The Table 8 shows that the present results agree with those of previous studies by Khalid and Adnan (2022) and Vishnu Ganesh et al. (2016).

**TABLE 8:** Comparison for $F^{\prime }\left(0\right)$ , $\alpha =5^{0},\phi =0$ .

$R_{e}$	Present	Vishnu Ganesh et al. (2016)	Alharbi and Adnan (2022)
20	−2.52719	−2.52719	−2.52719
40	−3.16971	−3.16971	−3.16971
60	−3.94214	−3.94214	−3.94214
80	−4.84507	−4.84507	−4.84507
100	−5.86916	−5.86916	−5.86916
120	−6.99705	−6.99705	−6.99705
140	−8.20733	−8.20733	−8.20733
160	−9.47855	−9.47855	−9.47855

## 8 Conclusion

In this paper, we have conducted a mathematical analysis of the velocity, heat transfer, and concentration profiles for nanofluids consisting of $\gamma {Al}_{2}O_{3}-H_{2}O$ , ${Al}_{2}O_{3}-H_{2}O$ , and $\gamma {Al}_{2}O_{3}-C_{2}H_{6}O_{2}$ between non-parallel walls in the presence of the magnetic field and thermal radiation. The used nanofluids’ governing partial differential equations are converted into nonlinear ordinary differential equations using the similarity transformation method. The HAM is used to solve the modeled equations. Important parameters were varied to analyze the behavior of velocity, temperature, and concentration fields. The following conclusions are drawn from the results.

• The velocity of the nanofluids decreases with an increase in the diverging parameter ( α>0 ), solid volume fraction (in the divergent channel), and the magnetic parameter. However, the greatest decrease in velocity is observed for ${Al}_{2}O_{3}-H_{2}O$ for all mentioned parameters.
• Velocities of the nanofluids $\gamma {Al}_{2}O_{3}-H_{2}O$ , ${Al}_{2}O_{3}-H_{2}O$ , and $\gamma {Al}_{2}O_{3}-C_{2}H_{6}O_{2}$ are enhanced by varying the convergent parameter α<0 and solid volume fraction (in the convergent channel).
• The temperature increases in all used nanofluids with an increase in the magnetic parameter, Eckert number, diverging parameter ( α>0 ), solid volume fraction (in the convergent channel), and thermal radiation (in the convergent channel).
• $\gamma {Al}_{2}O_{3}-C_{2}H_{6}O_{2}$ is observed to have better heat transfer performance than ${Al}_{2}O_{3}-H_{2}O$ and $\gamma {Al}_{2}O_{3}-H_{2}O$ in the presence of the magnetic field and thermal radiation.
• The temperature decreases in all used nanofluids by varying the convergent parameter ( α<0 ), solid volume fraction (in the divergent channel), and thermal radiation (in the divergent channel)
• The concentration field in the used nanofluids increases with an increase in the chemical reaction parameter and Schmidt number.
• Cf increases with an increase in Re, M, and diverging parameter ( α>0 ) for the nanofluids used.
• The Nusselt number increases with the increase in the diverging parameter α>0 and Eckert number (Ec) in all used nanofluids.
• The Sherwood number for nanofluids increases with an increase in the chemical reaction parameter ( $\left.K_{1}\right)$ and Schmidt number (Sc).

## Data Availability

The original contributions presented in the study are included in the article/supplementary material; further inquiries can be directed to the corresponding authors.

## Nomenclature

### Symbol

α: Converging/diverging parameter ( rad )
α<0: Converging parameter (*rad*)
θ: Polar angle (*NA*)
u,v,w: Components of velocities along axes ( m/s )
Sh: Sherwood number ( NA )
$T_{\infty }$: Ambient temperature ( °C or K )
$\mu_{nf}$: Nanofluid dynamic viscosity (Pascal-second (Pa s))
$\nu_{nf}$: Kinematic viscosity of nanofluid ( m2/s )
Ec: Eckert number ( NA )
$\rho_{nf}$: Density of nanofluid ( kg/m3 )
Pr: Prandtl number ( NA )
$B_{0}$: Uniform magnetic field ( $T\;\left(Tesla\right)$ )
$K_{1}$: Chemical reaction parameter (*NA*)
$\sigma_{nf}$: Electric conductivity of nanofluid ( sm )
Rd: Radiation parameter ( NA )
**R**: Radial direction
Sc: Schmidt number ( NA )
α>0: Diverging parameter ( rad )
Nu: Nusselt number
T: Fluid temperature ( °C or K )
$k_{nf}$: Thermal conductivity of nanofluid ( $W/\left(m\cdot K\right)$ )
$c_{p}$: Specific heat capacity ( $\left({kg}^{-1}K^{-1}\right)$ )
$k_{f}$: Thermal conductivity of base fluid ( $W/\left(m\cdot K\right)$ )
$T_{w}$: Temperature at the wall ( °C or K )
Re: Reynolds number ( NA )
c: Positive constant ( NA )
ϕ: Volume fraction ( NA )
$F\left(\eta \right)$: Dimensionless velocity ( NA )
$\theta \left(\eta \right)$: Dimensionless temperature ( NA )
$\gamma \left(\eta \right)$: Dimensionless concentration ( NA )
**M**: Magnetic parameter ( NA )
$C_{f}$: Coefficient of skin friction ( NA )
